# Chemometric Study of Fatty Acid Composition of Virgin Olive Oil from Four Widespread Greek Cultivars

**DOI:** 10.3390/molecules26144151

**Published:** 2021-07-08

**Authors:** Panagiota-Kyriaki Revelou, Marinos Xagoraris, Athanasia Alexandropoulou, Charalabos D. Kanakis, George K. Papadopoulos, Christos S. Pappas, Petros A. Tarantilis

**Affiliations:** 1 Laboratory of Chemistry, Department of Food Science and Human Nutrition, Agricultural University of Athens, 75 Iera Odos, 11855 Athens, Greece; p.revelou@aua.gr (P.-K.R.); mxagor@aua.gr (M.X.); chkanakis@aua.gr (C.D.K.); chrispap@aua.gr (C.S.P.); 2 Erganal Food and Environmental Testing Laboratories, Nikita 10, 18531 Piraeus, Greece; siaal22@yahoo.gr; 3 Laboratory of Plant Breeding and Biometry, Department of Crop Science, Agricultural University of Athens, 75 Iera Odos, 11855 Athens, Greece; gpapadop@aua.gr

**Keywords:** discriminant analysis, PCA, correlation, Koroneiki, Megaritiki, Amfissis, Manaki

## Abstract

Virgin olive oil (VOO) is one of the key components of the Mediterranean diet owing to the presence of monounsaturated fatty acids and various bioactive compounds. These beneficial traits, which are usually associated with the cultivar genotype, are highlighting the demand of identifying characteristics of olive oil that will ensure its authenticity. In this work, the fatty acid (FA) composition of 199 VOO samples from Koroneiki, Megaritiki, Amfissis, and Manaki cultivars was determined and studied by chemometrics. Olive cultivar greatly influenced the FA composition, namely, oleic acid (from 75.36% for Amfissis to 65.81% for Megaritiki) and linoleic acid (from 13.35% for Manaki to 6.70% for Koroneiki). Spearman’s *rho* correlation coefficients revealed differences and similarities among the olive oil cultivars. The use of the forward stepwise algorithm identified the FAs arachidonic acid, gadoleic acid, linoleic acid, α-linolenic acid, palmitoleic acid, and palmitic acid as the most significant for the differentiation of samples. The application of linear and quadratic cross-validation discriminant analysis resulted in the correct classification of 100.00% and 99.37% of samples, respectively. The findings demonstrated the special characteristics of the VOO samples derived from the four cultivars and their successful botanical differentiation based on FA composition.

## 1. Introduction

Virgin olive oil (VOO) is the sole edible olive oil which is extracted from the fruits of *Olea europaea* L. without having recourse to refining processes [1]. Olive oil has established hypolipidemic and antioxidant properties and its regular integration into the diet is said to result in major health benefits, including fewer cardiovascular diseases and neurological issues, and lower rates of breast and colon cancer [2]. Researchers have argued that these benefits stem from one of two features of olive oil: its high monounsaturated fatty acids (MUFA) content, where the main component is oleic acid, and the existence of minor molecules, for example, phytosterols, carotenoids, tocopherols, and polyphenols [3]. Oleic acid is the preponderant MUFA component (55.00–83.00%). Other fatty acids (FA) in olive oil are linoleic acid (2.50 to 21.00%), palmitic acid (7.50 to 20.00%), and α-linolenic acid (≤1.00%). Palmitoleic acid (0.30–3.50%) and gadoleic acid (≤0.50%) is also present, albeit in smaller proportions [4]. It has long been argued that human health is improved if saturated fats are replaced by monounsaturated fats since this lowers levels of cholesterol in the blood [5]. Olive oil is therefore a key element in the human diet.

Nevertheless, there is no stable and unvarying proportion of FA in olive oil, since this fluctuates according to cultivar genotypes, and edaphoclimatic factors [6,7]. FA profiles are shaped to a large extent by the cultivar which produces the olive oil [8,9,10]. In terms of genetic diversity, monovarietal olive oils, produced from a specific cultivar, have particular physical and biochemical traits and attributes that result in distinctive compositions and performances. As a result, both the health benefits and nutritional gains which are ascribed to olive oil, along with its sensory qualities, can vary significantly, since these are determined by its FA composition and the amount of other small compounds they contain [11]. Choosing cultivars which can be relied upon to have high levels of oleic acid could lead to the development of a range of products that stand out, from the nutritional and technological perspectives.

The Koroneiki variety (*Olea europaea* var. *microcarpa alba*) is the most widespread cultivar in Greece and can be found in many areas, including the Peloponnese and Crete. The Manaki cultivar (*Olea europaea* var. *minor rotunda*) can be found widely in the Peloponnese, particularly in Corinthia, Argolis, and Arcadia. In Central Greece, the dominant cultivars are Megaritiki (*Olea europaea* var. *argentata*) and Amfissis or Konservolia (*Olea europaea* var. *med. rotunda*). Megaritiki is the dominant cultivar in the Attica region but is also widespread in the Peloponnese. Amfissis can also be found in Thessaly, mainly in the Magnesia region [12,13].

Fats and oils, including olive oil, are ranked third in the 2018 EU food fraud report on non-compliances per product category [14]. Greece is, according to Eurostat, the EU’s fourth largest exporter of olive oil [15]. Hence, it is essential to ensure that the quality of Greek olive oil is checked and monitored in order to comply with the quality standards. Olive oil is a high-added-value product, and consumers need to be assured that the olive oil of Greek origin is authentic and accurately labeled [16]. Greek food producers, suppliers, and service industries will all benefit from quality control, and this will in turn solidify their position in the national and international marketplace.

Olive oils have been frequently classified according to the cultivar of origin based on their fatty acid composition [17,18,19,20,21,22]. In a previous work [23] the volatile profile of olive oil samples by solid phase microextraction—gas chromatography—mass spectrometry along with Fourier transform—infrared spectroscopy combined with discriminant analysis showed considerable potential for the classification of olive oil according to cultivar. The current research work sets out to evaluate the FA composition of 199 VOO samples from four widespread Greek olive oil cultivars, to explore the differences among the cultivars and to use chemometric methods to discover distinguishing FA in order to establish the authenticity of the botanical basis of Greek VOOs.

## 2. Results and Discussion

### 2.1. Conventional Quality Parameters

All the quality parameters tested (Table 1) conformed to those described in Commission Regulation (EEC) No 2568/91 [24] for extra virgin olive oil (EVOO) and VOO. Free acidity ranged from 0.41 ± 0.26 to 0.56 ± 0.44, within the limits of ≤0.8 and ≤2.0 for EVOO and VOO, respectively. The peroxide values (meq O_2_/kg oil) of samples ranged from 13.32 ± 4.94 to 17.29 ± 2.69 meq O_2_/kg oil are in accordance with the limits for both EVOOs and VOOs (≤20). Limits for the parameters K_232_, K_270_, and ∆K are ≤2.50, ≤0.22, and ≤0.01 for the EVOO, and ≤2.60, ≤0.25, and ≤0.01 for VOO [24]. Determination of spectroscopic values of the samples resulted in K_232_ from 1.97 ± 0.36 to 2.28 ± 0.32, K_270_ from 0.12 ± 0.03 to 0.16a ± 0.08, and ∆K 0.00 ± 0.00 for all samples analyzed, classifying samples as VOOs. The refractive index of olive oil samples was 1.469 ± 0.000.

The above quality parameters were subjected to analysis of variance (ANOVA) and post-hoc tests. A Kruskal–Wallis test was also performed, as in some cases small deviations from normality were observed. However, these deviations were insignificant, and therefore the results of the parametric post-hoc test Tukey HSD are reported in Table 1. No statistically significant differences based on quality parameters were observed between the samples, except for the refractive index. This indicates the low contribution of the conventional quality parameters in the differentiation of VOO samples among the four cultivars. The results are in accordance with the study of Kosma et al. [19] where the botanical classification of EVOO samples based on quality parameters was not feasible.

### 2.2. Fatty Acid Composition

Analysis of FA showed the presence of eleven FA (Table 2), with a variance in composition as a function of botanical origin [20]. The % mean values of FA were in accordance with the limits of the international olive oil council [4].

The dominant FA was oleic acid (C18:1) with the highest concentrations observed in Amfissis (75.36 ± 1.14%) and Koroneiki (74.70 ± 1.58%) VOO samples. Lower levels were detected in Megaritiki (65.81 ± 2.75%) and Manaki (70.15 ± 1.72%) cultivars. Adversely, for the polyunsaturated FA, linoleic acid (C18:2), the highest levels were observed in the Manaki (13.35 ± 1.07%) and Megaritiki (12.51 ± 2.02%) cultivars. The major saturated FAs were palmitic acid (C16:0), determined in the highest concentration in Megaritiki (15.53 ± 1.01%), and stearic acid (C18:0) in Koroneiki (2.78 ± 0.28%) and Manaki (2.63 ± 0.15%) samples. Interestingly, the highest levels of oleic/linoleic acid (C_18:1_/C_18:2_) and MUFA/PUFA ratios were observed in the Koroneiki cultivar which indicates a high performance against oxidative deterioration [25].

Similar results have been reported by previous studies for Koroneiki cultivar [18,22]. In a study from Stefanoudaki et al. [20] in Koroneiki olive oil samples from different maturity stages and producing areas of Crete the levels of oleic acid (18:1) (74.66–79.34%), and linoleic acid (18:2) (5.05–7.02%) were slightly different. Psomiadou et al. [26] studied 52 VOO samples from the Koroneiki cultivar and reported that oleic acid (C18:1) concentration ranged from 74.6% to 79.4% and 4.7% to 8.9% for linoleic acid (18:2). Limited research exists for the FA composition of olive oil from Amfissis, Manaki, and Megaritiki cultivars. Andreou et al. [27] evaluated the shelf-life evaluation of VOO from Amfissis, Manaki, and Tsounati cultivars extracted by non-thermal pretreatments. The oleic acid (18:1) and linoleic acid (18:2) levels using the traditional olive oil extraction technology were 70.68% and 12.66% for Amfissis, and for Manaki VOO samples 75.47% and 6.19%, respectively. In another study by Kosma et al. [19] the concentration of oleic acid (18:1) in Manaki extra virgin olive oil samples was slightly lower (72.93%). Iconomou et al. [28] and Katsoyannos et al. [29] studied the quality characteristics of Megaritiki olive oil and reported concentrations of 62.29% to 62.96% for oleic acid (18:1) and 11.64–14.39% for linoleic acid (18:2).

ANOVA and subsequent post-hoc tests between FA in association with botanical origin were performed (Table 2). Statistically significant differences were observed among the VOOs based on the levels of palmitoleic acid (16:1) and gadoleic acid (20:1), indicating a strong contribution on the discrimination between the four cultivars. Other FA such as arachidonic acid (20:4) and linoleic acid (18:2) were also significant for the differentiation of VOOs from Koroneiki, Megaritiki, and Amfissis cultivars. From the obtained results it is evident that a further application of chemometric methods to FA composition could be successful for the classification of samples with regard to cultivar.

### 2.3. Correlation Coefficients

**Figure 1:**
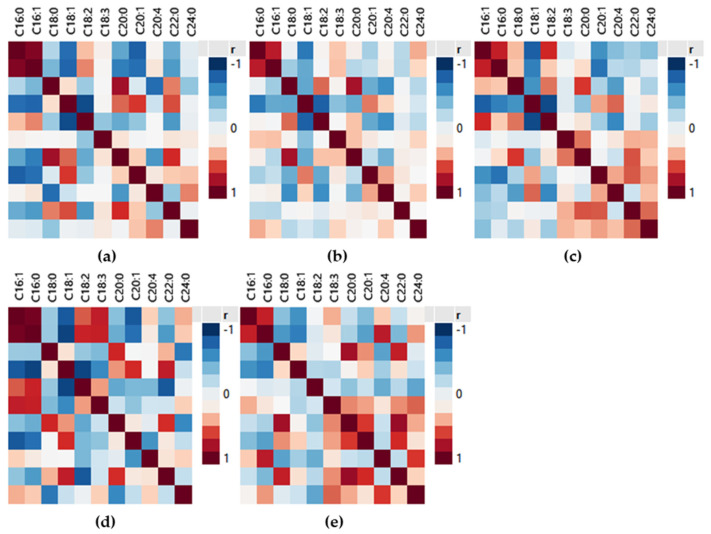
Color map on correlations from (**a**) the four VOO cultivars, (**b**) Koroneiki, (**c**) Megaritiki, (**d**) Manaki, (**e**) Amfissis.

Correlations of FA concentrations from the combination of the four olive oil cultivars as well as in each cultivar separately (Figure 1) were studied using Spearman’s rho correlation coefficients to designate potent similarities and differences between the cultivars. From the combined FA results of the four cultivars, strong positive correlations (*p* ≤ 0.01) were observed (Figure 1a) between palmitic acid (16:0)—palmitoleic acid (16:1), arachidic acid (20:0)—behenic acid (22:0), and arachidic acid (20:0)—stearic acid (18:0), while strong negative correlations were observed between gadoleic acid (20:1)—palmitoleic acid (16:1) and gadoleic acid (20:1)—palmitic acid (16:0). A medium to strong antagonistic relationships was detected among oleic acid (18:1)—linoleic acid (18:2), oleic acid (18:1)—palmitoleic acid (16:1), and oleic acid (18:1)—stearic acid (18:0) (Table S1 in Supplementary Materials). The correlations between palmitic acid (16:0)—palmitoleic acid (16:1) and arachidic acid (20:0)—stearic acid (18:0) have been previously reported from Stefanoudaki et al. [20] and Kritioti et al. [21] who studied olive oil samples from Mastoides, Koroneiki, and Cypriot (ladoelia) cultivars. The antagonistic relationship between oleic acid (18:1)—stearic acid (18:0) has also been reported by Kritioti et al. [21].

The above results can be explained by the kinetic differences of transformations taking place during the FA biosynthesis and the activity of FA desaturases during the maturation of olives. The oleic acid is converted to linoleic acid in an inverse association where the increased levels of one FA will result in the decrease of the other. Specifically, the FA are synthesized from the malonyl-acyl carrier protein with the subsequent action of the enzymatic system of FA synthase that mainly catalyzes the synthesis of palmitate. Consequently, the first FA produced from FA biosynthesis is palmitic acid (16:0) which can be elongated to stearic acid (18:0). Then through the catalyzing activity of stearoyl-ACP Δ9-desaturase, oleate desaturase, and linoleate desaturase, the oleic acid, linoleic acid, and α-linolenic acid, are formed, respectively [30,31,32].

A high degree of positive correlation coefficients were observed between palmitic (16:0)—palmitoleic acid (16:1) for all cultivars, especially in Manaki VOO samples (Figure 1d). Differences were observed in arachidic acid (20:0)—behenic acid (22:0), medium to strong correlations were observed in Megaritiki and Amfissis, while in Koroneiki and Manaki were low positive (Tables S2–S5). Positive correlations of arachidic acid (20:0)—stearic acid (18:0) were detected in all cultivars except from Manaki where the significance level was above 0.01. Strong negative correlations were observed between gadoleic acid (20:1)—palmitoleic acid (16:1) in Manaki and Megaritiki while in Koroneiki and Amfissis the correlations were insignificant. Strong negative correlations were also observed in gadoleic acid (20:1)—palmitic acid (16:0) in all cultivars except for Koroneiki samples. Medium to strong negative correlations of oleic acid (18:1)—palmitoleic acid (16:1) were detected in all cultivars. Strong antagonistic relationships were detected among oleic acid (18:1)—linoleic acid (18:2) in all cultivars except Amfissis, and medium antagonistic relationships were among oleic acid (18:1)—stearic acid (18:0) for Koroneiki and Megaritiki VOO samples. Summarizing the above results, common correlation patterns were detected in the four cultivars for the FA palmitic acid (16:0)—palmitoleic acid (16:1) and oleic acid (18:1)—palmitoleic acid (16:1), while several differences were observed among the remaining FA of this study depicting the differences in each cultivar.

### 2.4. Principal Component Analysis

**Figure 2:**
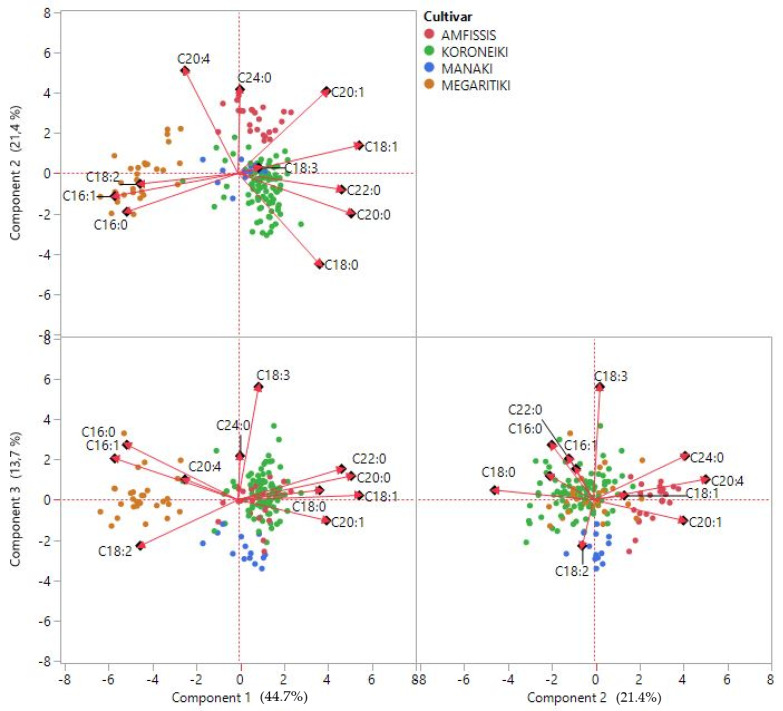
Principal component analysis (PCA) of VOO samples.

FAs were subjected to principal component analysis (PCA) in order to examine the possible grouping of VOO samples according to botanical origin. The plot of the first (component 1), second (component 2), and third principal component (component 3) is shown in Figure 2. The first axis accounted for 44.7% of the variance, the second 21.4%, and the third 13.7%, making a total of 79.8% of the variance for the three axes. A high negative correlation between arachidonic (20:4) and stearic acid (18:0) was observed based on the first two principal components, whereas a strong positive correlation between palmitic (16:0)—palmitoleic acid (16:1) was evident based on component 1–component 2 and component 1–component 3, confirming the Spearman’s rho correlation coefficients reported earlier in this study. 

**Figure 3:**
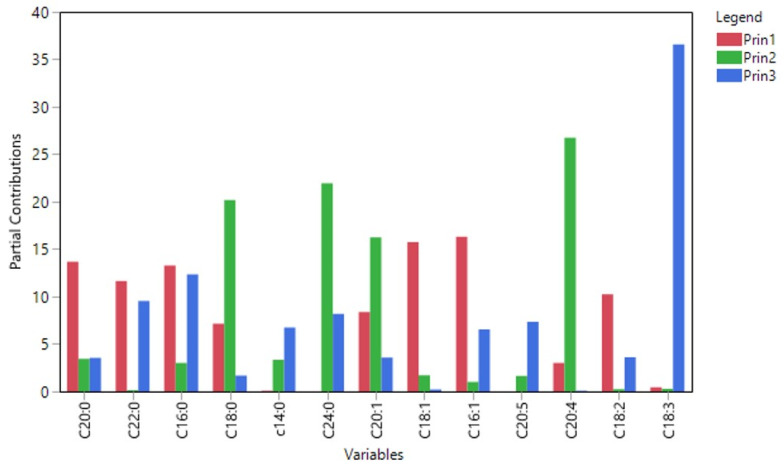
Partial contribution of fatty acids in the first three principal components.

The variables that contributed primarily in component 1 (Figure 3) were palmitic (16:0), palmitoleic (16:1), oleic (18:1), stearic (18:0), gadoleic (20:1), arachidic (20:0), behenic (22:0), and linoleic acid (18:2), with the highest contribution observed from palmitoleic (16:1) and oleic (18:1) acid. Arachidonic acid (20:4) mainly contributed to the formation of the second axis, followed by stearic (18:0), lignoceric (24:0), and gadoleic acid (20:1). α-linolenic acid (18:3) had a major contribution in the third component (Figure 3), palmitic (16:0), linoleic acid (18:2), and lignoceric acid (24:0). Based on the first two principal components a good separation has been achieved for the Megaritiki and Amfissis cultivars. Thus, the FA that participate in the formation of the two axes are successfully contributing to the differentiation of the VOO samples from Megaritiki and Amfissis. Nevertheless, VOO samples from the Manaki cultivar were not clearly separated from the Koroneiki samples. Consequently, other chemometric methods should be applied for their differentiation. 

### 2.5. Discriminant Analysis

Following the non-satisfactory results of PCA, further attempts towards the discrimination of VOOs was carried out using the supervised method of discriminant analysis (DA). The forward stepwise selection algorithm was applied for the determination of the most significant FA for the botanical differentiation of samples. The forward stepwise selection process was initiated with the addition of the variable with the highest contribution in the model. The next variable was added to the model if the entry probability was higher than the entry threshold value. After the addition of the third variable, the impact of removing each variable present in the model after its addition was evaluated. If the probability was higher than the removal threshold value, then the variable was removed from the model [33]. The discriminating power of each variable was defined by the F value. A high F value denotes great discriminating potential of the variable. 

Six FAs, namely, palmitoleic (C16:1), linoleic (C18:2), arachidonic (C20:4), α-linolenic (C18:3), gadoleic (C20:1), and palmitic acid (C16:0), were selected by the forward stepwise process as the most significant for the discrimination of VOOs (Table 3). Linear discriminant analysis (LDA) was performed using the six FAs selected by the algorithm. The values of Wilks’ Lambda = 0.006, F = 123.447, *p* < 0.0001 < 0.05 and Pillai’s trace = 2.331, F = 87.714, *p* < 0.0001 < 0.05, indicated significant differences among the means vectors of the VOOS from four olive oil cultivars.

According to the Eigenvalues and Bartlett’s statistic (Table 4), the three discriminant functions were significant in the differentiation of samples. However, Box’s test of Fisher’s F asymptotic approximation (−(2Log(M) = 242.457, *p* < 0.0001 < 0.05) denoted differences among the within-class covariance matrices which may be attributed to the different harvesting years of VOO samples.

**Figure 4:**
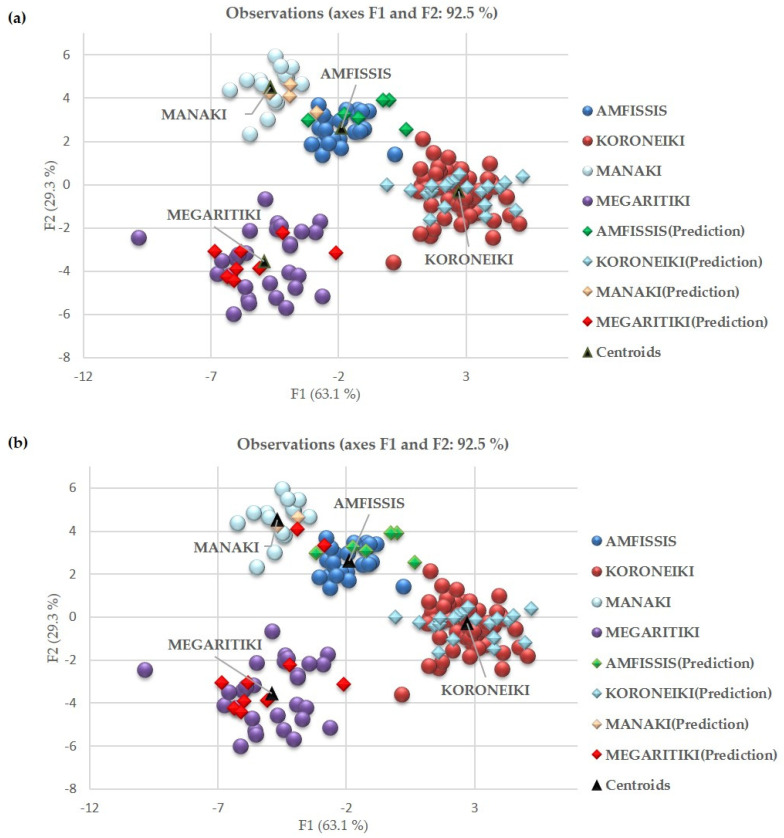
Botanical differentiation of VOO samples from the Koroneiki, Megaritiki, Amfissis, and Manaki cultivars using the stepwise variable selection and the classification methods: (**a**) LDA and (**b**) QDA.

The separation among the VOO samples from the four cultivars based on the first two discriminant functions F1 and F2 of the LDA model is presented in Figure 4a. The discriminant function F1, which accounts for 63.1% of the total variance, distinctly differentiates Megaritiki and Manaki from Koroneiki VOO samples, while Amfissis samples are not clearly differentiated. From the standardized canonical discriminant function coefficients (Table 5) we observed that the FA with the highest contribution based on F1 are linoleic (18:2), arachidonic (20:4), and α-linolenic acid (18:3). According to the discriminant function F2, palmitoleic (16:1), gadoleic (20:1), and linoleic acid (18:2) were the most significant FA for the discrimination of Manaki and Amfissis from Megaritiki VOO samples. The evaluation of the three discriminant functions for the mean points for each cultivar is presented at Table S6.

The correct classification for the training set of 158 VOOs was 100.00%. The LDA chemometric model was validated using the leave one out cross-validation (LOOCV), which enables to observe the prediction for a certain observation if it is left out of the training set. In order to acquire more realistic results about the model performance, an independent prediction set of 41 VOOs was utilized to further confirm the robustness of the model. The LOOCV rate obtained from the LDA model was 100.00%. Regarding the prediction set performance, one sample from the Amfissis cultivar was misclassified as Megaritiki, obtaining a 97.56% percentage of correct classification. 

Although LDA is generally robust to the violation of Box’s test criterion, quadratic discriminant analysis (QDA) is usually performed instead, where the within-class covariance matrices are assumed to be different [33]. Classification functions for LDA and QDA are given at Tables S7 and S8. These functions may be used to determine which class VOO sample is to be assigned to using values taken for the various explanatory variables. Each VOO sample is assigned to the class with the highest classification function. Discriminant functions used in the classification of samples for the training set are given at Tables S9 and S10 for LDA and QDA. Classification functions used to prediction for the prediction set are given at Tables S11 and S12, for LDA and QDA. The classification and prediction results of discriminant analysis were based on the classification function of each group and the discriminant functions. The discriminant functions were created from the training set and were not group-specific. This means that a sample was given a score based on the discriminant functions and was assigned to the group with the highest probability for group membership. Consequently, the samples of the training set were distinguished based on the discriminant functions. On the other hand, the samples of the prediction set, and any new observation, were classified based on the classification functions where the new observations will receive a discriminant score based on each group-specific function.

When QDA analysis was applied (Figure 4b), the same values for Wilks’ Lambda and Pillai’s trace were obtained, while correct classification, cross-validation, and prediction set rate were 99.37%, 98.10%, and 92.68%, respectively. Confusion matrix for the cross-validation and the prediction results of LDA and QDA are given at Tables S13 and S14. Regarding the prediction set performance, two VOO samples from Manaki and one VOO sample from the Amfissis cultivar were misclassified as Megaritiki. The percentage of prediction set is slightly lower from the cross-validation score for both LDA and QDA models. This observation may be attributed to the fact that the 41 samples of the prediction set were not included in the training set that was used for model building. On the contrary, the LOOCV was performed using the same samples that were used for model building. From the above results we concluded that LDA performed better than QDA for the differentiation of VOO samples according to cultivar.

Discrimination between the VOOs from the cultivars Koroneiki, Megaritiki, Amfissis, and Manaki based on FA has not been previously reported, though discrimination studies of other Greek olive oil cultivars have been performed based on FA composition with satisfactory results. Kosma et al. [19] determined nine FA and differentiated 74 samples from Manaki, Kolovi, Hontrolia, and Koutsourelia cultivars using LDA with a correct classification and cross-validation rate of 94.6% and 90.5%, respectively. In another study by Kosma et al. [18] 104 olive oil samples from the Koroneiki, Galano, Ladolia of Corfu, Adramitiani of Lesvos island, Athinolia of Laconia, and Samothraki cultivars were discriminated using MANOVA/LDA with a score of 94% and 92.1% for cross-validation using nine FAs. In our study, except for cross-validation, an external test set was also used to confirm the results of the developed chemometric models. Furthermore, in the above-mentioned studies, no specific FA are reported for the differentiation of olive oil with respect to cultivar. The findings of the current work indicate that characteristic FA can be identified and used in chemometric models in order to distinguish the botanical origin of olive oil.

## 3. Materials and Methods

### 3.1. Virgin Olive Oil Samples

A total of 199 VOO samples were collected from local producers and olive oil mills participating in the research program QuaAuthentic_GR, during the 2017–2018, 2018–2019, and 2019–2020 harvesting periods. The sample set included VOOs obtained by four single-cultivars: Koroneiki (115 samples), Megaritiki (35 samples), Amfissis (31 samples), and Manaki (18 samples). Sampling was carried out during November throughout the end of January. Olives were picked by hand or collected in nets at the stage of optimum maturity (maturity index 5–6) and processed in selected local olive mills by traditional three-phase system technology with a crusher. Samples were stored in dark glass bottles of 500 mL. The analyses of olive oil samples were performed soon after olive oil production.

### 3.2. Determination of Free Acidity, Peroxide Value and UV Absorption Coefficients

The quality indices including the refractive index were determined according to the official method of the Commission Regulation (EEC) No 2568/91 [24]. Free acidity was expressed as % oleic acid, peroxide value expressed as meq O_2_/kg, and specific extinction (K_232_ and K_270_) and its variation, ΔK, calculated from the absorption at 232–270 nm. 

### 3.3. Determination of Fatty Acids

Fatty acid methyl esters (FAME) were prepared based on the official method of the Commission Regulation (EEC) No 2568/91 [24]. Methyl esters were prepared by vigorous shaking of the VOO solution in hexane (0.1 g in 5 mL) with 0.5 mL of 2 N methanolic KOH in a screwcap vial. The analysis was performed by gas chromatography using a Perkin Elmer Clarus 500 chromatograph (Perkin Elmer, Waltham, MA, USA) equipped with a flame ionization detector. The column used was Supelco SP-2560 capillary column (75 m × 0.18 mm id × 0.14 μm film thickness) (Supelco, Bellefonte, PA, USA). The carrier gas was helium with a flow rate of 1.5 mL/min. An injection volume of 1 μL was used. The injector was operated in split mode (20:1 split ratio) at 250 °C. The column was maintained at 140 °C held for 5 min, heated to 170 °C at a rate of 8 °C/min, heated to 210 °C at a rate of 2 °C/min held for 2 min, heated to 250 °C at a rate of 20 °C/min and held to 250 °C for 10 min. The FA were identified from their retention times, using a FAME standard mixture (Sigma-Aldrich, St. Louis, MO, USA). The content of each FA was expressed as a percentage m/m from the peak area. Fatty acids identified in this study were C16:0, palmitic acid (hexadecanoic acid); C16:1ω-7, palmitoleic acid (*cis*-9-hexadecenoic acid); C18:0, stearic acid (octadecanoic acid); C18:1ω-9, oleic acid (*cis*-9-octadecenoic acid); C18:2ω-6, linoleic acid (*cis*,*cis*-9,12-octadecadienoic acid); C18:3ω-3, α-linolenic acid (*all*-*cis*-9,12,15-octadecatrienoic acid); C20:0, arachidic acid (eicosanoic acid); C20:1ω-11, gadoleic acid (*cis*-11-eicosenoic acid); 20:4ω-6, arachidonic acid (*all*-*cis*-5,8,11,14-eicosatetraenoic acid); C22:0, behenic acid (docosanoic acid); 24:0, lignoceric acid (tetracosanoic acid); MUFA, monounsaturated fatty acids; PUFA, polyunsaturated fatty acids; SFA, saturated fatty acids.

### 3.4. Statistical Analysis

Before statistical analysis data were standardized using the XLSTAT ver. 2020.3.1.0 software (Addinsoft Deutschland, Andernach, Germany). JMP statistical software version 13.0 (SAS Institute Inc., Cary, NC, USA) was used for the determination of Spearman’s *rho* correlation coefficients and the Principal Component Analysis. ANOVA, Kruskal–Wallis, and post-hoc comparisons were performed applying the Tukey HSD and Dunn test with Bonferroni correction using the XLSTAT ver. 2020.3.1.0 software (Addinsoft Deutschland, Andernach, Germany). The same software was used for the development of Discriminant Analysis models. One hundred and fifty-eight VOO samples were used as a training set (92 Koroneiki, 28 Megaritiki, 24 Amfissis, 14 Manaki) and 41 (23 Koroneiki, 7 Megaritiki, 7 Amfissis, 4 Manaki) as an external prediction set. The chemometric models were validated using the leave one out cross-validation method.

## 4. Conclusions

In the present work, the fatty acid composition of 199 VOO samples belonging to the cultivars of Koroneiki, Megaritiki, Amfissis, and Manaki was determined and studied by chemometrics. The elevated levels of MUFA observed in Amfissis (76.37%), and Koroneiki (75.81%) samples, as well as the high MUFA/PUFA ratio (9.77% for Koroneiki and 8.06% for Amfissis), suggested high oxidative stability of the virgin olive oils derived from both cultivars. ANOVA post-hoc tests revealed significant differences mainly among the fatty acids palmitoleic acid (16:1), gadoleic acid (20:1), arachidonic acid (20:4), and linoleic acid (18:2) indicating their potent contribution on the classification of samples with respect to cultivar. Spearman’s *rho* correlation coefficients between fatty acids highlighted both differences and similarities within the olive oil cultivars possibly attributed to the enzymes regulating the fatty acid biosynthesis. The application of principal components analysis did not effectively separated Koroneiki and Manaki olive oil samples though samples derived from Amfissis and Megaritiki were more clearly classified. The fatty acids, arachidonic acid (20:4), gadoleic acid (20:1), linoleic acid (18:2), α-linolenic acid (18:3), palmitoleic acid (16:1), and palmitic acid (16:0) were identified from the forward stepwise algorithm as the most significant for the botanical differentiation of VOOs. From the subsequent application of linear and quadratic discriminant analysis, 100.00% and 99.37% correct classification of samples was achieved, correspondingly. The chemometric models were validated by cross-validation and an external prediction set which was utilized to confirm the model’s robustness, providing a rate of 100.00% and 98.10% for the cross-validation, whereas the percentage obtained from the prediction set was 97.56% and 92.68%, for linear and quadratic discriminant analysis respectively. The current study accentuates the distinctive characteristics of Greek VOO associated with the fatty acid composition, while concurrently contributes to the efforts towards the characterization and authentication of Greek olive oil.

## Figures and Tables

**Table 1:** Mean values and standard deviation (SD) of conventional quality indices of VOO samples from Koroneiki, Megaritiki, Amfissis, and Manaki cultivars.

	Koroneiki		Megaritiki		Amfissis		Manaki	
	Mean	±SD	Mean	±SD	Mean	±SD	Mean	±SD
Acidity (% oleic acid)	0.56 ^a^	0.44	0.46 ^a^	0.41	0.41 ^a^	0.26	0.53 ^a^	0.15
PV (meqO_2_/Kg)	14.49 ^a^	5.35	13.32 ^a^	4.94	13.80 ^a^	5.07	17.29 ^a^	2.69
K_232_	1.97 ^a^	0.36	2.16 ^a^	0.41	2.16 ^a^	0.40	2.28 ^a^	0.32
K_270_	0.16 ^a^	0.08	0.14 ^a^	0.06	0.15 ^a^	0.06	0.12 ^a^	0.03
∆K	0.00 ^a^	0.00	0.00 ^a^	0.00	0.00 ^a^	0.00	0.00 ^a^	0.00
Refractive index	1.469 ^a^	0.000	1.469 ^ab^	0.000	1.469 ^bc^	0.000	1.469 ^c^	0.000

Means with different letters in the same row are significantly different (Tukey’s HSD test *p* < 0.05).

**Table 2:** Mean values and standard deviation (SD) of fatty acid composition (%) of VOO samples from Koroneiki, Megaritiki, Amfissis, and Manaki cultivars.

	Koroneiki		Megaritiki		Amfissis		Manaki	
	Mean	±SD	Mean	±SD	Mean	±SD	Mean	±SD
Palmitic acid (C16:0)	12.74 ^a^	0.79	15.53 ^b^	1.01	11.35 ^c^	0.68	11.25 ^c^	0.74
Palmitoleic acid (C16:1)	0.83 ^a^	0.12	1.53 ^b^	0.19	0.68 ^c^	0.09	0.56 ^d^	0.13
Stearic acid (C18:0)	2.78 ^a^	0.28	2.23 ^b^	0.14	2.30 ^b^	0.13	2.63 ^a^	0.15
Oleic acid (C18:1)	74.70 ^a^	1.58	65.81 ^b^	2.75	75.36 ^a^	1.14	70.15 ^c^	1.72
Linoleic acid (C18:2)	6.70 ^a^	1.08	12.51 ^b^	2.02	7.84 ^c^	0.65	13.35 ^b^	1.07
α-Linolenic acid (C18:3)	0.73 ^a^	0.07	0.70 ^a^	0.08	0.73 ^a^	0.06	0.58 ^b^	0.04
Arachidic acid (C20:0)	0.46 ^a^	0.02	0.38 ^b^	0.02	0.43 ^c^	0.02	0.45 ^ac^	0.01
Gadoleic acid (C20:1)	0.28 ^a^	0.02	0.23 ^b^	0.03	0.34 ^c^	0.00	0.30 ^d^	0.02
Arachidonic acid (C20:4)	0.47 ^a^	0.14	0.79 ^b^	0.19	0.94 ^c^	0.17	0.46 ^a^	0.07
Behenic acid (C22:0)	0.14 ^a^	0.02	0.11 ^b^	0.01	0.13 ^c^	0.01	0.13 ^c^	0.01
Lignoceric acid (C24:0)	0.06 ^a^	0.01	0.06 ^a^	0.01	0.07 ^b^	0.01	0.06 ^ab^	0.01
Σ^SFAs^	16.17 ^a^	0.85	18.30 ^b^	1.08	14.27 ^c^	0.62	14.52 ^c^	0.70
Σ^MUFAs^	75.81 ^a^	1.54	67.58 ^b^	2.60	76.37 ^a^	1.09	71.01 ^c^	1.62
Σ^PUFAs^	7.91 ^a^	1.02	13.99 ^b^	1.93	9.51 ^c^	0.59	14.39 ^b^	1.06
MUFA/PUFA	9.77 ^a^	1.45	4.96 ^b^	0.96	8.06 ^c^	0.53	4.97 ^b^	0.44
C_18:1_/C_18:2_	11.48 ^a^	2.17	5.46 ^b^	1.29	9.67 ^c^	0.81	5.29 ^b^	0.51

Means with different letters in the same row are significantly different (Tukey’s HSD test *p* < 0.05).

**Table 3:** List of the most discriminating variables according to the forward stepwise algorithm used in LDA and QDA classification.

Fatty Acids	Partial *R*²	*F*	Pr > *F*	Wilks’ Lambda	Pr < Lambda
C16:1	0.840	268.739	<0.0001	0.160	<0.0001
C18:2	0.792	194.597	<0.0001	0.033	<0.0001
C20:4	0.737	141.958	<0.0001	0.009	<0.0001
C18:3	0.171	10.384	<0.0001	0.007	<0.0001
C20:1	0.158	9.411	<0.0001	0.006	<0.0001
C16:0	0.089	4.853	0.003	0.006	<0.0001

**Table 4:** Canonical discriminant characteristics for the three discriminant functions (F1, F2, and F3) from the LDA and QDA classification.

Function	Eigenvalues	Bartlett’s Statistic	*p*	Discrimination (%)	Cumulative (%)	Canonical Correlation
1	11.285	788.999	0.000	63.137	63.137	0.958
2	5.249	407.727	0.000	29.368	92.505	0.917
3	1.340	129.198	0.000	7.495	100.000	0.757

**Table 5:** Standardized canonical discriminant function coefficients for the three discriminant functions (F1, F2, and F3) from the LDA and QDA classification.

Fatty Acids	F1	F2	F3
C20:4	−0.939	0.113	−0.658
C20:1	−0.142	0.434	−0.078
C18:2	−1.193	0.447	0.302
C18:3	0.401	−0.055	−0.334
C16:1	−0.173	−0.931	−0.493
C16:0	0.199	0.084	0.602

## Data Availability

Not applicable.

## Supplementary Materials

The following are available online, Table S1: Spearman’s *rho* correlation coefficients of fatty acid concentrations from the combination of the four olive oil cultivars, Table S2: Spearman’s rho correlation coefficients of fatty acid concentrations from the Koroneiki cultivar, Table S3: Spearman’s *rho* correlation coefficients of fatty acid concentrations from the Megaritiki cultivar, Table S4: Spearman’s *rho* correlation coefficients of fatty acid concentrations from the Manaki cultivar, Table S5: Spearman’s *rho* correlation coefficients of fatty acid concentrations from the Amfissis cultivar, Table S6: Functions at the centroids for linear and quadratic discriminant analysis, Table S7: Classification functions for linear discriminant analysis, Table S8: Classification functions for quadratic discriminant analysis, Table S9: Prior and posterior classification, membership probabilities and discriminant function scores of the training set for linear discriminant analysis, Table S10: Prior and posterior classification, membership probabilities and discriminant function scores of the training set for quadratic discriminant analysis, Table S11: Results for the prediction sample set from linear discriminant analysis, Table S12: Results for the prediction samples of quadratic discriminant analysis, Table S13: Confusion matrix for the cross-validation results of linear and quadratic discriminant analysis, Table S14: Confusion matrix for the prediction results of linear and quadratic discriminant analysis.

## Sample Availability

Not available.
