# Supplementary material for: Chemometric Study of Fatty Acid Composition of Virgin Olive Oil from Four Widespread Greek Cultivars
Source: Molecules. 2021 Jul 8;26(14):4151. doi: 10.3390/molecules26144151 (PMC8303967; doi:10.3390/molecules26144151)
Supplement: Supplementary file 1 [file molecules-26-04151-s001.zip › molecules-1276985-supplementary.pdf]

Supplementary Materials

Chemometric Study of Fatty Acid Composition of Virgin Olive Oil from Four Widespread Greek Cultivars

Panagiota-Kyriaki Revelou [1], Marinos Xagoraris [1], Athanasia Alexandropoulou[2], Charalabos D. Kanakis [1], George K. Papadopoulos[3], Christos S. Pappas [1] and Petros A. Tarantilis [1,*]

Table S1. Spearman's *rho* correlation coefficients of fatty acid concentrations from the cultivar.

Variables	C16:0	C16:1	C18:0	C18:1	C18:2	C18:3	C20:0	C20:1	C20:4	C22:0	C24:0
C16:0	**1**	**0.899**	-0.139	**-0.603**	0.148	0.177	**-0.250**	**-0.745**	0.120	**-0.319**	-0.082
C16:1	**0.899**	**1**	**-0.284**	**-0.491**	0.116	**0.226**	**-0.397**	**-0.668**	0.184	**-0.337**	-0.089
C18:0	-0.139	**-0.284**	**1**	0.143	**-0.309**	0.077	**0.839**	-0.121	**-0.777**	**0.633**	**-0.351**
C18:1	**-0.603**	**-0.491**	0.143	**1**	**-0.789**	0.128	**0.213**	**0.525**	-0.037	**0.548**	0.025
C18:2	0.148	0.116	**-0.309**	**-0.789**	**1**	**-0.264**	**-0.345**	**-0.218**	0.139	**-0.630**	-0.008
C18:3	0.177	**0.226**	0.077	0.128	**-0.264**	**1**	**0.287**	0.101	0.099	**0.317**	**0.344**
C20:0	**-0.250**	**-0.397**	**0.839**	**0.213**	**-0.345**	**0.287**	**1**	0.174	**-0.574**	**0.715**	0.018
C20:1	**-0.745**	**-0.668**	-0.121	**0.525**	**-0.218**	0.101	0.174	**1**	0.197	**0.232**	**0.448**
C20:4	0.120	0.184	**-0.777**	-0.037	0.139	0.099	**-0.574**	0.197	**1**	**-0.480**	**0.512**
C22:0	**-0.319**	**-0.337**	**0.633**	**0.548**	**-0.630**	**0.317**	**0.715**	**0.232**	**-0.480**	**1**	-0.055
C24:0	-0.082	-0.089	**-0.351**	0.025	-0.008	**0.344**	0.018	**0.448**	**0.512**	-0.055	**1**

Values in bold are different from 0 with a significance level $p \leq 0.01$.

Table S2. Spearman's *rho* correlation coefficients of fatty acid concentrations from the Koroneiki.

Variables	C16:0	C16:1	C18:0	C18:1	C18:2	C18:3	C20:0	C20:1	C20:4	C22:0	C24:0
C16:0	**1**	**0.717**	0.087	**-0.673**	0.059	**0.325**	0.262	-0.232	0.154	**-0.289**	**0.418**
C16:1	**0.717**	**1**	**-0.288**	**-0.401**	-0.051	**0.380**	-0.144	-0.017	0.231	**-0.359**	**0.381**
C18:0	0.087	**-0.288**	**1**	**-0.499**	**0.490**	0.090	**0.780**	**-0.469**	**-0.594**	0.123	-0.219
C18:1	**-0.673**	**-0.401**	**-0.499**	**1**	**-0.708**	**-0.298**	**-0.525**	**0.484**	**0.336**	0.136	-0.077
C18:2	0.059	-0.051	**0.490**	**-0.708**	**1**	0.062	**0.335**	**-0.426**	**-0.608**	-0.050	**-0.344**
C18:3	**0.325**	**0.380**	0.090	**-0.298**	0.062	**1**	**0.347**	0.212	0.057	0.214	**0.461**
C20:0	0.262	-0.144	**0.780**	**-0.525**	**0.335**	**0.347**	**1**	-0.242	**-0.402**	**0.356**	0.156
C20:1	-0.232	-0.017	**-0.469**	**0.484**	**-0.426**	0.212	-0.242	**1**	**0.391**	0.264	0.245
C20:4	0.154	0.231	**-0.594**	**0.336**	**-0.608**	0.057	**-0.402**	**0.391**	**1**	-0.053	**0.451**
C22:0	**-0.289**	**-0.359**	0.123	0.136	-0.050	0.214	**0.356**	0.264	-0.053	**1**	0.153
C24:0	**0.418**	**0.381**	-0.219	-0.077	**-0.344**	**0.461**	0.156	0.245	**0.451**	0.153	**1**

Values in bold are different from 0 with a significance level $p \leq 0.01$.

Table S3. Spearman's *rho* correlation coefficients of fatty acid concentrations from the Megaritiki cultivar.

Variables	C16:1	C16:0	C18:0	C18:1	C18:2	C18:3	C20:0	C20:1	C20:4	C22:0	C24:0
C16:1	**1**	**0.680**	0.348	**-0.692**	**0.524**	-0.109	-0.048	**-0.640**	-0.220	-0.363	-0.397
C16:0	**0.680**	**1**	0.338	**-0.635**	0.239	-0.083	0.308	**-0.718**	-0.055	-0.249	-0.055
C18:0	0.348	0.338	**1**	**-0.503**	0.445	-0.016	**0.529**	-0.415	**-0.539**	0.075	-0.248
C18:1	**-0.692**	**-0.635**	**-0.503**	**1**	**-0.801**	-0.057	-0.318	**0.501**	0.408	0.131	0.210
C18:2	**0.524**	0.239	0.445	**-0.801**	**1**	0.274	0.216	-0.260	**-0.540**	-0.042	-0.223
C18:3	-0.109	-0.083	-0.016	-0.057	0.274	**1**	**0.571**	0.343	0.127	0.483	0.416
C20:0	-0.048	0.308	**0.529**	-0.318	0.216	**0.571**	**1**	0.008	-0.137	**0.515**	0.338
C20:1	**-0.640**	**-0.718**	-0.415	**0.501**	-0.260	0.343	0.008	**1**	0.405	**0.509**	**0.515**
C20:4	-0.220	-0.055	**-0.539**	0.408	**-0.540**	0.127	-0.137	0.405	**1**	-0.148	0.335
C22:0	-0.363	-0.249	0.075	0.131	-0.042	0.483	**0.515**	**0.509**	-0.148	**1**	**0.526**
C24:0	-0.397	-0.055	-0.248	0.210	-0.223	0.416	0.338	**0.515**	0.335	**0.526**	**1**

Values in bold are different from 0 with a significance level $p \leq 0.01$.

Table S4. Spearman's *rho* correlation coefficients of fatty acid concentrations from the Manaki cultivar.

Variables	C16:1	C16:0	C18:0	C18:1	C18:2	C18:3	C20:0	C20:1	C20:4	C22:0	C24:0
C16:1	**1**	**0.950**	-0.275	**-0.878**	0.506	**0.799**	-0.266	**-0.819**	0.359	-0.353	0.411
C16:0	**0.950**	**1**	-0.181	**-0.873**	0.486	**0.754**	-0.278	**-0.714**	0.317	-0.453	0.344
C18:0	-0.275	-0.181	**1**	0.145	-0.344	-0.348	0.572	0.091	-0.025	0.185	**-0.722**
C18:1	**-0.878**	**-0.873**	0.145	**1**	**-0.741**	-0.623	0.345	**0.788**	-0.146	0.665	-0.211
C18:2	0.506	0.486	-0.344	**-0.741**	**1**	0.404	-0.400	-0.512	-0.262	**-0.731**	0.067
C18:3	**0.799**	**0.754**	-0.348	-0.623	0.404	**1**	-0.037	-0.414	-0.090	-0.170	0.342
C20:0	-0.266	-0.278	0.572	0.345	-0.400	-0.037	**1**	0.319	-0.193	0.415	-0.580
C20:1	**-0.819**	**-0.714**	0.091	**0.788**	-0.512	-0.414	0.319	**1**	-0.531	0.324	-0.250
C20:4	0.359	0.317	-0.025	-0.146	-0.262	-0.090	-0.193	-0.531	**1**	0.231	0.325
C22:0	-0.353	-0.453	0.185	0.665	**-0.731**	-0.170	0.415	0.324	0.231	**1**	-0.083
C24:0	0.411	0.344	**-0.722**	-0.211	0.067	0.342	-0.580	-0.250	0.325	-0.083	**1**

Values in bold are different from 0 with a significance level $p \leq 0.01$.

Table S5. Spearman's *rho* correlation coefficients of fatty acid concentrations from the Amfissis cultivar.

Variables	C16:1	C16:0	C18:0	C18:1	C18:2	C18:3	C20:0	C20:1	C20:4	C22:0	C24:0
C16:1	**1**	**0.689**	-0.319	**-0.566**	0.007	0.377	-0.087	-0.413	0.410	-0.142	0.129
C16:0	**0.689**	**1**	**-0.590**	**-0.614**	-0.223	0.123	-0.307	**-0.582**	**0.790**	-0.469	0.357
C18:0	-0.319	**-0.590**	**1**	0.360	-0.035	0.196	**0.784**	0.478	**-0.541**	**0.737**	-0.062
C18:1	**-0.566**	**-0.614**	0.360	**1**	-0.311	-0.241	0.117	0.326	-0.284	0.263	-0.072
C18:2	0.007	-0.223	-0.035	-0.311	**1**	-0.138	-0.272	-0.061	-0.510	-0.140	**-0.598**
C18:3	0.377	0.123	0.196	-0.241	-0.138	**1**	**0.560**	0.416	0.246	0.502	0.460
C20:0	-0.087	-0.307	**0.784**	0.117	-0.272	**0.560**	**1**	0.502	-0.144	**0.867**	0.373
C20:1	-0.413	**-0.582**	0.478	0.326	-0.061	0.416	0.502	**1**	-0.320	**0.621**	0.187
C20:4	0.410	**0.790**	**-0.541**	-0.284	-0.510	0.246	-0.144	-0.320	**1**	-0.232	**0.588**
C22:0	-0.142	-0.469	**0.737**	0.263	-0.140	0.502	**0.867**	**0.621**	-0.232	**1**	0.296
C24:0	0.129	0.357	-0.062	-0.072	**-0.598**	0.460	0.373	0.187	**0.588**	0.296	**1**

Values in bold are different from 0 with a significance level $p \leq 0.01$.

Table S6. Functions at the centroids for linear and quadratic discriminant analysis.

	F1	F2	F3
AMFISSIS	-1.887	2.611	-2.264
KORONEIKI	2.699	-0.292	0.224
MANAKI	-4.681	4.520	2.370
MEGARITIKI	-4.911	-3.540	0.021

Table S7. Classification functions for linear discriminant analysis.

	AMFISSIS	KORONEIKI	MANAKI	MEGARITIKI
Intercept	-9.637	-4.251	-26.402	-20.057
C20:4	5.691	-4.347	5.360	6.725
C20:1	2.864	-0.956	4.427	-1.529
C18:2	6.343	-7.612	19.295	9.928
C18:3	-0.170	1.201	-3.421	-2.089
C16:1	-2.446	-0.754	-11.296	10.221
C16:0	-2.950	1.255	1.703	-2.446

Table S8. Classification functions for quadratic discriminant analysis.

	AMFISSIS	KORONEIKI	MANAKI	MEGARITIKI
Intercept	-19.432	-0.533	-153.410	-8.557
C20:4	15.449	-4.753	172.484	6.048
C20:1	11.814	-2.206	100.012	-1.370
C18:2	15.406	-10.701	195.494	5.171
C18:3	-3.689	1.533	21.997	-1.489
C16:1	8.491	-3.278	151.140	2.483
C16:0	-19.861	1.855	-211.305	0.879
C20:4*C20:4	-4.034	-2.693	-64.279	-1.967
C20:4*C20:1	1.232	1.849	-64.470	1.642
C20:4*C18:2	-8.760	-4.079	-115.554	-3.392
C20:4*C18:3	0.496	-0.267	-24.139	0.879
C20:4*C16:1	-5.587	1.084	-65.503	1.470
C20:4*C16:0	11.993	1.461	112.239	0.790
C20:1*C20:1	-10.143	-3.654	-39.144	-1.942
C20:1*C18:2	-1.514	-2.747	-67.691	1.297
C20:1*C18:3	6.453	1.713	5.676	0.009
C20:1*C16:1	-8.139	-0.931	-135.433	-0.872
C20:1*C16:0	-9.607	-2.516	100.148	-3.154
C18:2*C18:2	-15.657	-6.535	-62.336	-3.523
C18:2*C18:3	0.330	1.090	-17.327	1.326
C18:2*C16:1	-1.664	-0.350	-103.292	5.447
C18:2*C16:0	9.094	0.941	138.342	-0.661
C18:3*C18:3	-2.149	-1.042	-9.026	-0.618
C18:3*C16:1	6.218	1.102	33.854	-1.230
C18:3*C16:0	1.374	1.057	4.053	0.661
C16:1*C16:1	-19.760	-8.630	-186.114	-5.355
C16:1*C16:0	17.742	8.120	251.891	4.630
C16:0*C16:0	-15.916	-4.992	-119.448	-4.038

Table S9. Prior and posterior classification, membership probabilities, and discriminant function scores of the training set for linear discriminant analysis.

Observation	Prior	Posterior	Pr(AMFISSIS)	Pr(KORONEIKI)	Pr(MANAKI)	Pr(MEGARITIKI)	F1	F2	F3
Obs1	KORONEIKI	KORONEIKI	0.000	1.000	0.000	0.000	2.906	0.400	-0.094
Obs2	KORONEIKI	KORONEIKI	0.000	1.000	0.000	0.000	2.272	0.399	0.450
Obs3	KORONEIKI	KORONEIKI	0.000	1.000	0.000	0.000	2.450	0.044	0.395
Obs4	KORONEIKI	KORONEIKI	0.000	1.000	0.000	0.000	2.753	0.026	-0.237
Obs5	KORONEIKI	KORONEIKI	0.000	1.000	0.000	0.000	1.612	-0.121	0.001
Obs6	KORONEIKI	KORONEIKI	0.000	1.000	0.000	0.000	4.564	-0.536	2.215
Obs7	KORONEIKI	KORONEIKI	0.000	1.000	0.000	0.000	4.572	-0.556	2.982
Obs8	KORONEIKI	KORONEIKI	0.000	1.000	0.000	0.000	1.231	-0.041	1.390
Obs9	KORONEIKI	KORONEIKI	0.000	1.000	0.000	0.000	2.666	-0.450	0.082
Obs10	KORONEIKI	KORONEIKI	0.000	1.000	0.000	0.000	2.868	-0.331	0.262
Obs11	KORONEIKI	KORONEIKI	0.000	1.000	0.000	0.000	2.138	-1.550	-1.027
Obs12	KORONEIKI	KORONEIKI	0.000	1.000	0.000	0.000	3.180	-0.218	0.699
Obs13	KORONEIKI	KORONEIKI	0.000	1.000	0.000	0.000	2.801	0.575	0.149
Obs14	KORONEIKI	KORONEIKI	0.000	1.000	0.000	0.000	2.546	0.377	0.365
Obs15	KORONEIKI	KORONEIKI	0.000	1.000	0.000	0.000	1.957	0.457	0.757
Obs16	KORONEIKI	KORONEIKI	0.104	0.896	0.000	0.000	1.276	2.137	-1.155
Obs17	KORONEIKI	KORONEIKI	0.000	1.000	0.000	0.000	2.432	-0.393	-0.116
Obs18	KORONEIKI	KORONEIKI	0.000	1.000	0.000	0.000	2.194	0.895	0.471
Obs19	KORONEIKI	KORONEIKI	0.000	1.000	0.000	0.000	2.335	0.292	0.074
Obs20	KORONEIKI	KORONEIKI	0.000	1.000	0.000	0.000	2.306	0.020	0.051
Obs21	KORONEIKI	KORONEIKI	0.000	1.000	0.000	0.000	2.539	0.434	-0.304
Obs22	KORONEIKI	KORONEIKI	0.000	1.000	0.000	0.000	2.156	0.529	-0.165
Obs23	KORONEIKI	KORONEIKI	0.000	1.000	0.000	0.000	2.479	0.328	-0.191
Obs24	KORONEIKI	KORONEIKI	0.000	1.000	0.000	0.000	2.718	0.047	-0.323

Obs25	KORO-NEIKI	KORO-NEIKI	0.000	1.000	0.000	0.000	2.290	0.750	0.279
Obs26	KORO-NEIKI	KORO-NEIKI	0.000	1.000	0.000	0.000	2.584	0.719	-0.556
Obs27	KORO-NEIKI	KORO-NEIKI	0.000	1.000	0.000	0.000	3.487	-0.646	-0.898
Obs28	KORO-NEIKI	KORO-NEIKI	0.000	1.000	0.000	0.000	3.341	-0.744	-1.020
Obs29	KORO-NEIKI	KORO-NEIKI	0.000	1.000	0.000	0.000	2.484	0.369	1.355
Obs30	KORO-NEIKI	KORO-NEIKI	0.000	1.000	0.000	0.000	3.134	-0.332	-0.176
Obs31	KORO-NEIKI	KORO-NEIKI	0.000	1.000	0.000	0.000	2.916	-0.405	-0.205
Obs32	KORO-NEIKI	KORO-NEIKI	0.000	1.000	0.000	0.000	2.919	-1.067	0.286
Obs33	KORO-NEIKI	KORO-NEIKI	0.000	1.000	0.000	0.000	2.783	-0.698	-0.551
Obs34	KORO-NEIKI	KORO-NEIKI	0.000	1.000	0.000	0.000	3.416	-0.619	-1.350
Obs35	KORO-NEIKI	KORO-NEIKI	0.000	1.000	0.000	0.000	2.276	-0.163	0.648
Obs36	KORO-NEIKI	KORO-NEIKI	0.000	1.000	0.000	0.000	1.701	-0.127	0.099
Obs37	KORO-NEIKI	KORO-NEIKI	0.000	1.000	0.000	0.000	2.685	-0.204	-0.120
Obs38	KORO-NEIKI	KORO-NEIKI	0.000	1.000	0.000	0.000	2.931	-0.901	-0.880
Obs39	KORO-NEIKI	KORO-NEIKI	0.000	1.000	0.000	0.000	1.946	-0.118	1.259
Obs40	KORO-NEIKI	KORO-NEIKI	0.000	1.000	0.000	0.000	4.049	-2.425	-1.005
Obs41	KORO-NEIKI	KORO-NEIKI	0.000	1.000	0.000	0.000	2.218	0.665	0.951
Obs42	KORO-NEIKI	KORO-NEIKI	0.000	1.000	0.000	0.000	1.960	0.615	0.918
Obs43	KORO-NEIKI	KORO-NEIKI	0.000	1.000	0.000	0.000	1.335	0.254	2.599
Obs44	KORO-NEIKI	KORO-NEIKI	0.000	1.000	0.000	0.000	1.205	0.720	2.388
Obs45	KORO-NEIKI	KORO-NEIKI	0.000	1.000	0.000	0.000	3.968	0.159	-0.265
Obs46	KORO-NEIKI	KORO-NEIKI	0.000	1.000	0.000	0.000	2.610	-0.042	1.159
Obs47	KORO-NEIKI	KORO-NEIKI	0.000	1.000	0.000	0.000	3.115	0.321	1.582
Obs48	KORO-NEIKI	KORO-NEIKI	0.000	1.000	0.000	0.000	2.922	-1.254	1.172
Obs49	KORO-NEIKI	KORO-NEIKI	0.000	1.000	0.000	0.000	3.585	-0.252	-0.618
Obs50	KORO-NEIKI	KORO-NEIKI	0.000	1.000	0.000	0.000	3.543	-0.333	-0.699

Obs51	KORO-NEIKI	KORO-NEIKI	0.000	1.000	0.000	0.000	3.477	-0.682	0.326
Obs52	KORO-NEIKI	KORO-NEIKI	0.000	1.000	0.000	0.000	1.901	0.850	1.846
Obs53	KORO-NEIKI	KORO-NEIKI	0.001	0.999	0.000	0.000	1.721	1.475	-0.771
Obs54	KORO-NEIKI	KORO-NEIKI	0.000	1.000	0.000	0.000	1.789	0.512	0.872
Obs55	KORO-NEIKI	KORO-NEIKI	0.000	1.000	0.000	0.000	2.818	-0.362	-0.887
Obs56	KORO-NEIKI	KORO-NEIKI	0.000	1.000	0.000	0.000	2.355	-0.243	0.045
Obs57	KORO-NEIKI	KORO-NEIKI	0.000	1.000	0.000	0.000	2.366	0.143	-0.175
Obs58	KORO-NEIKI	KORO-NEIKI	0.000	1.000	0.000	0.000	3.715	-0.413	0.566
Obs59	KORO-NEIKI	KORO-NEIKI	0.000	1.000	0.000	0.000	2.648	-0.189	1.567
Obs60	KORO-NEIKI	KORO-NEIKI	0.000	1.000	0.000	0.000	3.245	-0.361	1.566
Obs61	KORO-NEIKI	KORO-NEIKI	0.000	1.000	0.000	0.000	3.174	-0.531	-0.322
Obs62	KORO-NEIKI	KORO-NEIKI	0.000	1.000	0.000	0.000	2.680	-0.132	-0.921
Obs63	KORO-NEIKI	KORO-NEIKI	0.000	1.000	0.000	0.000	2.797	0.739	-1.172
Obs64	KORO-NEIKI	KORO-NEIKI	0.000	1.000	0.000	0.000	1.616	-0.951	0.719
Obs65	KORO-NEIKI	KORO-NEIKI	0.000	0.996	0.000	0.004	0.144	-3.583	0.768
Obs66	KORO-NEIKI	KORO-NEIKI	0.000	1.000	0.000	0.000	2.280	-0.694	-0.783
Obs67	KORO-NEIKI	KORO-NEIKI	0.000	1.000	0.000	0.000	3.396	-1.292	-1.925
Obs68	KORO-NEIKI	KORO-NEIKI	0.000	1.000	0.000	0.000	1.422	-0.188	1.412
Obs69	KORO-NEIKI	KORO-NEIKI	0.000	1.000	0.000	0.000	3.035	-0.473	0.488
Obs70	KORO-NEIKI	KORO-NEIKI	0.000	1.000	0.000	0.000	2.632	-0.920	0.363
Obs71	KORO-NEIKI	KORO-NEIKI	0.000	1.000	0.000	0.000	2.750	0.110	-0.437
Obs72	KORO-NEIKI	KORO-NEIKI	0.000	1.000	0.000	0.000	2.801	-0.562	-1.080
Obs73	KORO-NEIKI	KORO-NEIKI	0.000	1.000	0.000	0.000	3.080	-0.809	-0.616
Obs74	KORO-NEIKI	KORO-NEIKI	0.000	1.000	0.000	0.000	3.128	-0.579	1.146
Obs75	KORO-NEIKI	KORO-NEIKI	0.000	1.000	0.000	0.000	1.130	-0.300	-0.148
Obs76	KORO-NEIKI	KORO-NEIKI	0.000	1.000	0.000	0.000	2.804	-0.954	-0.087

Obs77	KORO-NEIKI	KORO-NEIKI	0.000	1.000	0.000	0.000	3.117	-1.455	-0.809
Obs78	KORO-NEIKI	KORO-NEIKI	0.000	1.000	0.000	0.000	1.604	-2.384	-0.225
Obs79	KORO-NEIKI	KORO-NEIKI	0.000	1.000	0.000	0.000	2.804	-1.834	-1.717
Obs80	KORO-NEIKI	KORO-NEIKI	0.000	1.000	0.000	0.000	3.398	-0.522	0.328
Obs81	KORO-NEIKI	KORO-NEIKI	0.000	1.000	0.000	0.000	4.107	-0.075	2.295
Obs82	KORO-NEIKI	KORO-NEIKI	0.000	1.000	0.000	0.000	3.927	0.990	1.427
Obs83	KORO-NEIKI	KORO-NEIKI	0.000	1.000	0.000	0.000	4.714	-1.405	1.154
Obs84	KORO-NEIKI	KORO-NEIKI	0.000	1.000	0.000	0.000	5.058	-1.794	1.359
Obs85	KORO-NEIKI	KORO-NEIKI	0.000	1.000	0.000	0.000	4.075	-1.648	0.333
Obs86	KORO-NEIKI	KORO-NEIKI	0.000	1.000	0.000	0.000	3.840	0.258	0.727
Obs87	KORO-NEIKI	KORO-NEIKI	0.000	1.000	0.000	0.000	2.941	-0.631	1.179
Obs88	KORO-NEIKI	KORO-NEIKI	0.000	1.000	0.000	0.000	3.496	-0.268	0.211
Obs89	KORO-NEIKI	KORO-NEIKI	0.000	1.000	0.000	0.000	2.293	1.284	-0.591
Obs90	KORO-NEIKI	KORO-NEIKI	0.000	1.000	0.000	0.000	1.722	-2.068	-1.419
Obs91	KORO-NEIKI	KORO-NEIKI	0.000	1.000	0.000	0.000	2.780	0.362	0.876
Obs92	KORO-NEIKI	KORO-NEIKI	0.000	1.000	0.000	0.000	1.196	-2.250	0.023
Obs93	MEGARI-TIKI	MEGARI-TIKI	0.008	0.000	0.000	0.992	-2.731	-1.709	-1.523
Obs94	MEGARI-TIKI	MEGARI-TIKI	0.000	0.000	0.000	1.000	-6.766	-4.106	-1.899
Obs95	MEGARI-TIKI	MEGARI-TIKI	0.000	0.000	0.000	1.000	-9.858	-2.429	-1.035
Obs96	MEGARI-TIKI	MEGARI-TIKI	0.001	0.000	0.000	0.999	-4.400	-1.750	-2.778
Obs97	MEGARI-TIKI	MEGARI-TIKI	0.000	0.000	0.000	1.000	-5.559	-5.280	0.764
Obs98	MEGARI-TIKI	MEGARI-TIKI	0.000	0.000	0.000	1.000	-2.638	-5.141	0.403
Obs99	MEGARI-TIKI	MEGARI-TIKI	0.000	0.000	0.000	1.000	-3.952	-4.047	1.264
Obs100	MEGARI-TIKI	MEGARI-TIKI	0.000	0.000	0.000	1.000	-5.498	-2.124	0.880
Obs101	MEGARI-TIKI	MEGARI-TIKI	0.000	0.000	0.000	1.000	-4.693	-4.563	0.072
Obs102	MEGARI-TIKI	MEGARI-TIKI	0.000	0.000	0.000	1.000	-6.131	-5.982	0.719

Obs103	MEGARI-TIKI	MEGARI-TIKI	0.000	0.000	0.000	1.000	-3.576	-4.201	-0.498
Obs104	MEGARI-TIKI	MEGARI-TIKI	0.005	0.000	0.000	0.995	-2.928	-2.204	-2.930
Obs105	MEGARI-TIKI	MEGARI-TIKI	0.000	0.000	0.000	1.000	-4.893	-0.639	0.179
Obs106	MEGARI-TIKI	MEGARI-TIKI	0.000	0.000	0.000	1.000	-6.289	-4.205	-1.588
Obs107	MEGARI-TIKI	MEGARI-TIKI	0.000	0.000	0.000	1.000	-6.030	-3.399	1.500
Obs108	MEGARI-TIKI	MEGARI-TIKI	0.000	0.000	0.000	1.000	-3.454	-2.154	-1.316
Obs109	MEGARI-TIKI	MEGARI-TIKI	0.000	0.000	0.000	1.000	-4.461	-5.235	-0.603
Obs110	MEGARI-TIKI	MEGARI-TIKI	0.000	0.000	0.000	1.000	-3.915	-2.710	0.249
Obs111	MEGARI-TIKI	MEGARI-TIKI	0.000	0.000	0.000	1.000	-4.435	-2.036	0.951
Obs112	MEGARI-TIKI	MEGARI-TIKI	0.000	0.000	0.000	1.000	-5.636	-3.162	0.995
Obs113	MEGARI-TIKI	MEGARI-TIKI	0.000	0.000	0.000	1.000	-6.574	-3.495	0.908
Obs114	MEGARI-TIKI	MEGARI-TIKI	0.000	0.000	0.000	1.000	-5.930	-3.225	0.502
Obs115	MEGARI-TIKI	MEGARI-TIKI	0.000	0.000	0.000	1.000	-4.288	-1.901	0.354
Obs116	MEGARI-TIKI	MEGARI-TIKI	0.000	0.000	0.000	1.000	-3.719	-4.747	0.797
Obs117	MEGARI-TIKI	MEGARI-TIKI	0.000	0.000	0.000	1.000	-5.520	-5.473	1.234
Obs118	MEGARI-TIKI	MEGARI-TIKI	0.000	0.000	0.000	1.000	-5.680	-4.717	0.567
Obs119	MEGARI-TIKI	MEGARI-TIKI	0.000	0.000	0.000	1.000	-4.062	-5.689	2.441
Obs120	MEGARI-TIKI	MEGARI-TIKI	0.000	0.000	0.000	1.000	-3.904	-2.798	-0.032
Obs121	AMFISSIS	AMFISSIS	1.000	0.000	0.000	0.000	-1.200	3.347	-2.996
Obs122	AMFISSIS	AMFISSIS	1.000	0.000	0.000	0.000	-1.246	3.473	-2.798
Obs123	AMFISSIS	AMFISSIS	1.000	0.000	0.000	0.000	-2.697	1.890	-2.069
Obs124	AMFISSIS	AMFISSIS	1.000	0.000	0.000	0.000	-2.482	2.357	-2.831
Obs125	AMFISSIS	AMFISSIS	1.000	0.000	0.000	0.000	-1.995	2.118	-3.051
Obs126	AMFISSIS	AMFISSIS	1.000	0.000	0.000	0.000	-2.228	2.132	-3.214
Obs127	AMFISSIS	AMFISSIS	1.000	0.000	0.000	0.000	-2.765	2.642	-2.474
Obs128	AMFISSIS	AMFISSIS	1.000	0.000	0.000	0.000	-2.457	2.964	-2.650
Obs129	AMFISSIS	AMFISSIS	1.000	0.000	0.000	0.000	-2.657	1.372	-2.903
Obs130	AMFISSIS	AMFISSIS	1.000	0.000	0.000	0.000	-2.533	2.545	-2.533
Obs131	AMFISSIS	AMFISSIS	1.000	0.000	0.000	0.000	-1.965	2.968	-2.545
Obs132	AMFISSIS	AMFISSIS	1.000	0.000	0.000	0.000	-2.624	3.237	-2.681
Obs133	AMFISSIS	AMFISSIS	1.000	0.000	0.000	0.000	-1.916	1.707	-2.660
Obs134	AMFISSIS	AMFISSIS	1.000	0.000	0.000	0.000	-2.407	1.928	-3.363
Obs135	AMFISSIS	AMFISSIS	0.999	0.000	0.001	0.000	-2.788	3.711	-1.133
Obs136	AMFISSIS	AMFISSIS	1.000	0.000	0.000	0.000	-3.064	1.861	-1.261
Obs137	AMFISSIS	AMFISSIS	1.000	0.000	0.000	0.000	-1.701	3.477	-1.916

Obs138	AMFISSIS	AMFISSIS	1.000	0.000	0.000	0.000	-1.264	3.185	-1.818
Obs139	AMFISSIS	AMFISSIS	1.000	0.000	0.000	0.000	-1.021	2.586	-3.017
Obs140	AMFISSIS	AMFISSIS	1.000	0.000	0.000	0.000	-1.386	2.430	-1.970
Obs141	AMFISSIS	AMFISSIS	0.938	0.062	0.000	0.000	0.210	1.428	-1.982
Obs142	AMFISSIS	AMFISSIS	1.000	0.000	0.000	0.000	-0.835	3.424	-0.590
Obs143	AMFISSIS	AMFISSIS	1.000	0.000	0.000	0.000	-1.104	3.397	-0.931
Obs144	AMFISSIS	AMFISSIS	1.000	0.000	0.000	0.000	-1.154	2.485	-0.962
Obs145	MANAKI	MANAKI	0.000	0.000	1.000	0.000	-3.469	4.653	1.943
Obs146	MANAKI	MANAKI	0.000	0.000	1.000	0.000	-5.100	4.840	2.240
Obs147	MANAKI	MANAKI	0.000	0.000	1.000	0.000	-4.048	4.965	1.956
Obs148	MANAKI	MANAKI	0.000	0.000	1.000	0.000	-5.017	4.613	2.312
Obs149	MANAKI	MANAKI	0.000	0.000	1.000	0.000	-4.427	3.796	1.604
Obs150	MANAKI	MANAKI	0.000	0.000	1.000	0.000	-4.801	3.007	2.246
Obs151	MANAKI	MANAKI	0.000	0.000	1.000	0.000	-4.097	5.037	2.456
Obs152	MANAKI	MANAKI	0.000	0.000	1.000	0.000	-6.268	4.387	3.266
Obs153	MANAKI	MANAKI	0.000	0.000	1.000	0.000	-3.870	5.457	2.416
Obs154	MANAKI	MANAKI	0.000	0.000	1.000	0.000	-4.502	5.941	2.805
Obs155	MANAKI	MANAKI	0.000	0.000	1.000	0.000	-4.290	5.498	2.527
Obs156	MANAKI	MANAKI	0.000	0.000	1.000	0.000	-5.625	4.838	3.580
Obs157	MANAKI	MANAKI	0.000	0.000	1.000	0.000	-5.494	2.341	1.973
Obs158	MANAKI	MANAKI	0.000	0.000	1.000	0.000	-4.525	3.904	1.850

Table S10. Prior and posterior classification, membership probabilities, and discriminant function scores of the training set for quadratic discriminant analysis.

Observation	Prior	Posterior	Pr(AMFISSIS)	Pr(KORONEIKI)	Pr(MANAKI)	Pr(MEGARITIKI)	F1	F2	F3
Obs1	KORONEIKI	KORONEIKI	0.000	1.000	0.000	0.000	2.906	0.400	-0.094
Obs2	KORONEIKI	KORONEIKI	0.000	1.000	0.000	0.000	2.272	0.399	0.450
Obs3	KORONEIKI	KORONEIKI	0.000	1.000	0.000	0.000	2.450	0.044	0.395
Obs4	KORONEIKI	KORONEIKI	0.000	1.000	0.000	0.000	2.753	0.026	-0.237
Obs5	KORONEIKI	KORONEIKI	0.000	1.000	0.000	0.000	1.612	-0.121	0.001
Obs6	KORONEIKI	KORONEIKI	0.000	1.000	0.000	0.000	4.564	-0.536	2.215
Obs7	KORONEIKI	KORONEIKI	0.000	1.000	0.000	0.000	4.572	-0.556	2.982
Obs8	KORONEIKI	KORONEIKI	0.000	1.000	0.000	0.000	1.231	-0.041	1.390
Obs9	KORONEIKI	KORONEIKI	0.000	1.000	0.000	0.000	2.666	-0.450	0.082
Obs10	KORONEIKI	KORONEIKI	0.000	1.000	0.000	0.000	2.868	-0.331	0.262
Obs11	KORONEIKI	KORONEIKI	0.000	1.000	0.000	0.000	2.138	-1.550	-1.027
Obs12	KORONEIKI	KORONEIKI	0.000	1.000	0.000	0.000	3.180	-0.218	0.699

Obs13	KORONEIKI	KORO-NEIKI	0.000	1.000	0.000	0.000	2.801	0.575	0.149
Obs14	KORONEIKI	KORO-NEIKI	0.000	1.000	0.000	0.000	2.546	0.377	0.365
Obs15	KORONEIKI	KORO-NEIKI	0.000	1.000	0.000	0.000	1.957	0.457	0.757
Obs16	KORONEIKI	KORO-NEIKI	0.000	1.000	0.000	0.000	1.276	2.137	-1.155
Obs17	KORONEIKI	KORO-NEIKI	0.000	1.000	0.000	0.000	2.432	-0.393	-0.116
Obs18	KORONEIKI	KORO-NEIKI	0.000	1.000	0.000	0.000	2.194	0.895	0.471
Obs19	KORONEIKI	KORO-NEIKI	0.000	1.000	0.000	0.000	2.335	0.292	0.074
Obs20	KORONEIKI	KORO-NEIKI	0.000	1.000	0.000	0.000	2.306	0.020	0.051
Obs21	KORONEIKI	KORO-NEIKI	0.000	1.000	0.000	0.000	2.539	0.434	-0.304
Obs22	KORONEIKI	KORO-NEIKI	0.000	1.000	0.000	0.000	2.156	0.529	-0.165
Obs23	KORONEIKI	KORO-NEIKI	0.000	1.000	0.000	0.000	2.479	0.328	-0.191
Obs24	KORONEIKI	KORO-NEIKI	0.000	1.000	0.000	0.000	2.718	0.047	-0.323
Obs25	KORONEIKI	KORO-NEIKI	0.000	1.000	0.000	0.000	2.290	0.750	0.279
Obs26	KORONEIKI	KORO-NEIKI	0.000	1.000	0.000	0.000	2.584	0.719	-0.556
Obs27	KORONEIKI	KORO-NEIKI	0.000	1.000	0.000	0.000	3.487	-0.646	-0.898
Obs28	KORONEIKI	KORO-NEIKI	0.000	1.000	0.000	0.000	3.341	-0.744	-1.020
Obs29	KORONEIKI	KORO-NEIKI	0.000	1.000	0.000	0.000	2.484	0.369	1.355
Obs30	KORONEIKI	KORO-NEIKI	0.000	1.000	0.000	0.000	3.134	-0.332	-0.176
Obs31	KORONEIKI	KORO-NEIKI	0.000	1.000	0.000	0.000	2.916	-0.405	-0.205
Obs32	KORONEIKI	KORO-NEIKI	0.000	1.000	0.000	0.000	2.919	-1.067	0.286
Obs33	KORONEIKI	KORO-NEIKI	0.000	1.000	0.000	0.000	2.783	-0.698	-0.551
Obs34	KORONEIKI	KORO-NEIKI	0.000	1.000	0.000	0.000	3.416	-0.619	-1.350
Obs35	KORONEIKI	KORO-NEIKI	0.000	1.000	0.000	0.000	2.276	-0.163	0.648
Obs36	KORONEIKI	KORO-NEIKI	0.000	1.000	0.000	0.000	1.701	-0.127	0.099
Obs37	KORONEIKI	KORO-NEIKI	0.000	1.000	0.000	0.000	2.685	-0.204	-0.120
Obs38	KORONEIKI	KORO-NEIKI	0.000	1.000	0.000	0.000	2.931	-0.901	-0.880

Obs39	KORONEIKI	KORO-NEIKI	0.000	1.000	0.000	0.000	1.946	-0.118	1.259
Obs40	KORONEIKI	KORO-NEIKI	0.000	1.000	0.000	0.000	4.049	-2.425	-1.005
Obs41	KORONEIKI	KORO-NEIKI	0.000	1.000	0.000	0.000	2.218	0.665	0.951
Obs42	KORONEIKI	KORO-NEIKI	0.000	1.000	0.000	0.000	1.960	0.615	0.918
Obs43	KORONEIKI	KORO-NEIKI	0.000	1.000	0.000	0.000	1.335	0.254	2.599
Obs44	KORONEIKI	KORO-NEIKI	0.000	1.000	0.000	0.000	1.205	0.720	2.388
Obs45	KORONEIKI	KORO-NEIKI	0.000	1.000	0.000	0.000	3.968	0.159	-0.265
Obs46	KORONEIKI	KORO-NEIKI	0.000	1.000	0.000	0.000	2.610	-0.042	1.159
Obs47	KORONEIKI	KORO-NEIKI	0.000	1.000	0.000	0.000	3.115	0.321	1.582
Obs48	KORONEIKI	KORO-NEIKI	0.000	1.000	0.000	0.000	2.922	-1.254	1.172
Obs49	KORONEIKI	KORO-NEIKI	0.000	1.000	0.000	0.000	3.585	-0.252	-0.618
Obs50	KORONEIKI	KORO-NEIKI	0.000	1.000	0.000	0.000	3.543	-0.333	-0.699
Obs51	KORONEIKI	KORO-NEIKI	0.000	1.000	0.000	0.000	3.477	-0.682	0.326
Obs52	KORONEIKI	KORO-NEIKI	0.000	1.000	0.000	0.000	1.901	0.850	1.846
Obs53	KORONEIKI	KORO-NEIKI	0.000	1.000	0.000	0.000	1.721	1.475	-0.771
Obs54	KORONEIKI	KORO-NEIKI	0.000	1.000	0.000	0.000	1.789	0.512	0.872
Obs55	KORONEIKI	KORO-NEIKI	0.000	1.000	0.000	0.000	2.818	-0.362	-0.887
Obs56	KORONEIKI	KORO-NEIKI	0.000	1.000	0.000	0.000	2.355	-0.243	0.045
Obs57	KORONEIKI	KORO-NEIKI	0.000	1.000	0.000	0.000	2.366	0.143	-0.175
Obs58	KORONEIKI	KORO-NEIKI	0.000	1.000	0.000	0.000	3.715	-0.413	0.566
Obs59	KORONEIKI	KORO-NEIKI	0.000	1.000	0.000	0.000	2.648	-0.189	1.567
Obs60	KORONEIKI	KORO-NEIKI	0.000	1.000	0.000	0.000	3.245	-0.361	1.566
Obs61	KORONEIKI	KORO-NEIKI	0.000	1.000	0.000	0.000	3.174	-0.531	-0.322
Obs62	KORONEIKI	KORO-NEIKI	0.000	1.000	0.000	0.000	2.680	-0.132	-0.921
Obs63	KORONEIKI	KORO-NEIKI	0.000	1.000	0.000	0.000	2.797	0.739	-1.172
Obs64	KORONEIKI	KORO-NEIKI	0.000	1.000	0.000	0.000	1.616	-0.951	0.719

Obs65	KORONEIKI	**MEGARI-TIKI**	0.000	0.056	0.000	0.944	0.144	-3.583	0.768
Obs66	KORONEIKI	KORO-NEIKI	0.000	1.000	0.000	0.000	2.280	-0.694	-0.783
Obs67	KORONEIKI	KORO-NEIKI	0.000	1.000	0.000	0.000	3.396	-1.292	-1.925
Obs68	KORONEIKI	KORO-NEIKI	0.000	1.000	0.000	0.000	1.422	-0.188	1.412
Obs69	KORONEIKI	KORO-NEIKI	0.000	1.000	0.000	0.000	3.035	-0.473	0.488
Obs70	KORONEIKI	KORO-NEIKI	0.000	1.000	0.000	0.000	2.632	-0.920	0.363
Obs71	KORONEIKI	KORO-NEIKI	0.000	1.000	0.000	0.000	2.750	0.110	-0.437
Obs72	KORONEIKI	KORO-NEIKI	0.000	1.000	0.000	0.000	2.801	-0.562	-1.080
Obs73	KORONEIKI	KORO-NEIKI	0.000	1.000	0.000	0.000	3.080	-0.809	-0.616
Obs74	KORONEIKI	KORO-NEIKI	0.000	1.000	0.000	0.000	3.128	-0.579	1.146
Obs75	KORONEIKI	KORO-NEIKI	0.000	1.000	0.000	0.000	1.130	-0.300	-0.148
Obs76	KORONEIKI	KORO-NEIKI	0.000	1.000	0.000	0.000	2.804	-0.954	-0.087
Obs77	KORONEIKI	KORO-NEIKI	0.000	1.000	0.000	0.000	3.117	-1.455	-0.809
Obs78	KORONEIKI	KORO-NEIKI	0.000	1.000	0.000	0.000	1.604	-2.384	-0.225
Obs79	KORONEIKI	KORO-NEIKI	0.000	1.000	0.000	0.000	2.804	-1.834	-1.717
Obs80	KORONEIKI	KORO-NEIKI	0.000	1.000	0.000	0.000	3.398	-0.522	0.328
Obs81	KORONEIKI	KORO-NEIKI	0.000	1.000	0.000	0.000	4.107	-0.075	2.295
Obs82	KORONEIKI	KORO-NEIKI	0.000	1.000	0.000	0.000	3.927	0.990	1.427
Obs83	KORONEIKI	KORO-NEIKI	0.000	1.000	0.000	0.000	4.714	-1.405	1.154
Obs84	KORONEIKI	KORO-NEIKI	0.000	1.000	0.000	0.000	5.058	-1.794	1.359
Obs85	KORONEIKI	KORO-NEIKI	0.000	1.000	0.000	0.000	4.075	-1.648	0.333
Obs86	KORONEIKI	KORO-NEIKI	0.000	1.000	0.000	0.000	3.840	0.258	0.727
Obs87	KORONEIKI	KORO-NEIKI	0.000	1.000	0.000	0.000	2.941	-0.631	1.179
Obs88	KORONEIKI	KORO-NEIKI	0.000	1.000	0.000	0.000	3.496	-0.268	0.211
Obs89	KORONEIKI	KORO-NEIKI	0.000	1.000	0.000	0.000	2.293	1.284	-0.591
Obs90	KORONEIKI	KORO-NEIKI	0.000	1.000	0.000	0.000	1.722	-2.068	-1.419

Obs91	KORONEIKI	KORO-NEIKI	0.000	1.000	0.000	0.000	2.780	0.362	0.876
Obs92	KORONEIKI	KORO-NEIKI	0.000	1.000	0.000	0.000	1.196	-2.250	0.023
Obs93	MEGARITIKI	MEGARI-TIKI	0.000	0.000	0.000	1.000	-2.731	-1.709	-1.523
Obs94	MEGARITIKI	MEGARI-TIKI	0.000	0.000	0.000	1.000	-6.766	-4.106	-1.899
Obs95	MEGARITIKI	MEGARI-TIKI	0.000	0.000	0.000	1.000	-9.858	-2.429	-1.035
Obs96	MEGARITIKI	MEGARI-TIKI	0.000	0.000	0.000	1.000	-4.400	-1.750	-2.778
Obs97	MEGARITIKI	MEGARI-TIKI	0.000	0.000	0.000	1.000	-5.559	-5.280	0.764
Obs98	MEGARITIKI	MEGARI-TIKI	0.000	0.000	0.000	1.000	-2.638	-5.141	0.403
Obs99	MEGARITIKI	MEGARI-TIKI	0.000	0.000	0.000	1.000	-3.952	-4.047	1.264
Obs100	MEGARITIKI	MEGARI-TIKI	0.000	0.000	0.000	1.000	-5.498	-2.124	0.880
Obs101	MEGARITIKI	MEGARI-TIKI	0.000	0.000	0.000	1.000	-4.693	-4.563	0.072
Obs102	MEGARITIKI	MEGARI-TIKI	0.000	0.000	0.000	1.000	-6.131	-5.982	0.719
Obs103	MEGARITIKI	MEGARI-TIKI	0.000	0.000	0.000	1.000	-3.576	-4.201	-0.498
Obs104	MEGARITIKI	MEGARI-TIKI	0.000	0.000	0.000	1.000	-2.928	-2.204	-2.930
Obs105	MEGARITIKI	MEGARI-TIKI	0.000	0.000	0.000	1.000	-4.893	-0.639	0.179
Obs106	MEGARITIKI	MEGARI-TIKI	0.000	0.000	0.000	1.000	-6.289	-4.205	-1.588
Obs107	MEGARITIKI	MEGARI-TIKI	0.000	0.000	0.000	1.000	-6.030	-3.399	1.500
Obs108	MEGARITIKI	MEGARI-TIKI	0.000	0.000	0.000	1.000	-3.454	-2.154	-1.316
Obs109	MEGARITIKI	MEGARI-TIKI	0.000	0.000	0.000	1.000	-4.461	-5.235	-0.603
Obs110	MEGARITIKI	MEGARI-TIKI	0.000	0.000	0.000	1.000	-3.915	-2.710	0.249
Obs111	MEGARITIKI	MEGARI-TIKI	0.000	0.000	0.000	1.000	-4.435	-2.036	0.951
Obs112	MEGARITIKI	MEGARI-TIKI	0.000	0.000	0.000	1.000	-5.636	-3.162	0.995
Obs113	MEGARITIKI	MEGARI-TIKI	0.000	0.000	0.000	1.000	-6.574	-3.495	0.908
Obs114	MEGARITIKI	MEGARI-TIKI	0.000	0.000	0.000	1.000	-5.930	-3.225	0.502
Obs115	MEGARITIKI	MEGARI-TIKI	0.000	0.000	0.000	1.000	-4.288	-1.901	0.354
Obs116	MEGARITIKI	MEGARI-TIKI	0.000	0.000	0.000	1.000	-3.719	-4.747	0.797

Obs117	MEGARITIKI	MEGARI-TIKI	0.000	0.000	0.000	1.000	-5.520	-5.473	1.234
Obs118	MEGARITIKI	MEGARI-TIKI	0.000	0.000	0.000	1.000	-5.680	-4.717	0.567
Obs119	MEGARITIKI	MEGARI-TIKI	0.000	0.000	0.000	1.000	-4.062	-5.689	2.441
Obs120	MEGARITIKI	MEGARI-TIKI	0.000	0.000	0.000	1.000	-3.904	-2.798	-0.032
Obs121	AMFISSIS	AMFISSIS	1.000	0.000	0.000	0.000	-1.200	3.347	-2.996
Obs122	AMFISSIS	AMFISSIS	1.000	0.000	0.000	0.000	-1.246	3.473	-2.798
Obs123	AMFISSIS	AMFISSIS	1.000	0.000	0.000	0.000	-2.697	1.890	-2.069
Obs124	AMFISSIS	AMFISSIS	1.000	0.000	0.000	0.000	-2.482	2.357	-2.831
Obs125	AMFISSIS	AMFISSIS	1.000	0.000	0.000	0.000	-1.995	2.118	-3.051
Obs126	AMFISSIS	AMFISSIS	1.000	0.000	0.000	0.000	-2.228	2.132	-3.214
Obs127	AMFISSIS	AMFISSIS	1.000	0.000	0.000	0.000	-2.765	2.642	-2.474
Obs128	AMFISSIS	AMFISSIS	1.000	0.000	0.000	0.000	-2.457	2.964	-2.650
Obs129	AMFISSIS	AMFISSIS	1.000	0.000	0.000	0.000	-2.657	1.372	-2.903
Obs130	AMFISSIS	AMFISSIS	1.000	0.000	0.000	0.000	-2.533	2.545	-2.533
Obs131	AMFISSIS	AMFISSIS	1.000	0.000	0.000	0.000	-1.965	2.968	-2.545
Obs132	AMFISSIS	AMFISSIS	1.000	0.000	0.000	0.000	-2.624	3.237	-2.681
Obs133	AMFISSIS	AMFISSIS	1.000	0.000	0.000	0.000	-1.916	1.707	-2.660
Obs134	AMFISSIS	AMFISSIS	1.000	0.000	0.000	0.000	-2.407	1.928	-3.363
Obs135	AMFISSIS	AMFISSIS	1.000	0.000	0.000	0.000	-2.788	3.711	-1.133
Obs136	AMFISSIS	AMFISSIS	1.000	0.000	0.000	0.000	-3.064	1.861	-1.261
Obs137	AMFISSIS	AMFISSIS	1.000	0.000	0.000	0.000	-1.701	3.477	-1.916
Obs138	AMFISSIS	AMFISSIS	1.000	0.000	0.000	0.000	-1.264	3.185	-1.818
Obs139	AMFISSIS	AMFISSIS	1.000	0.000	0.000	0.000	-1.021	2.586	-3.017
Obs140	AMFISSIS	AMFISSIS	1.000	0.000	0.000	0.000	-1.386	2.430	-1.970
Obs141	AMFISSIS	AMFISSIS	0.997	0.003	0.000	0.000	0.210	1.428	-1.982
Obs142	AMFISSIS	AMFISSIS	1.000	0.000	0.000	0.000	-0.835	3.424	-0.590
Obs143	AMFISSIS	AMFISSIS	1.000	0.000	0.000	0.000	-1.104	3.397	-0.931
Obs144	AMFISSIS	AMFISSIS	1.000	0.000	0.000	0.000	-1.154	2.485	-0.962
Obs145	MANAKI	MANAKI	0.000	0.000	1.000	0.000	-3.469	4.653	1.943
Obs146	MANAKI	MANAKI	0.000	0.000	1.000	0.000	-5.100	4.840	2.240
Obs147	MANAKI	MANAKI	0.000	0.000	1.000	0.000	-4.048	4.965	1.956
Obs148	MANAKI	MANAKI	0.000	0.000	1.000	0.000	-5.017	4.613	2.312
Obs149	MANAKI	MANAKI	0.000	0.000	1.000	0.000	-4.427	3.796	1.604
Obs150	MANAKI	MANAKI	0.000	0.000	1.000	0.000	-4.801	3.007	2.246
Obs151	MANAKI	MANAKI	0.000	0.000	1.000	0.000	-4.097	5.037	2.456
Obs152	MANAKI	MANAKI	0.000	0.000	1.000	0.000	-6.268	4.387	3.266
Obs153	MANAKI	MANAKI	0.000	0.000	1.000	0.000	-3.870	5.457	2.416
Obs154	MANAKI	MANAKI	0.000	0.000	1.000	0.000	-4.502	5.941	2.805
Obs155	MANAKI	MANAKI	0.000	0.000	1.000	0.000	-4.290	5.498	2.527
Obs156	MANAKI	MANAKI	0.000	0.000	1.000	0.000	-5.625	4.838	3.580
Obs157	MANAKI	MANAKI	0.000	0.000	1.000	0.000	-5.494	2.341	1.973
Obs158	MANAKI	MANAKI	0.000	0.000	1.000	0.000	-4.525	3.904	1.850

Table S11. Results for the prediction sample set from linear discriminant analysis.

	Predicted class	Pr(AMFIS-SIS)	Pr(KORO-NEIKI)	Pr(MANAKI)	Pr(MEGARI-TIKI)	F1	F2	F3
PredObs1	MANAKI	0.000	0.000	1.000	0.000	-3.910	4.088	1.579
PredObs2	MANAKI	0.000	0.000	1.000	0.000	-4.695	4.220	3.079
PredObs3	MANAKI	0.000	0.000	1.000	0.000	-2.851	3.326	2.466
PredObs4	MANAKI	0.000	0.000	1.000	0.000	-3.889	4.613	2.921
PredObs5	KORONEIKI	0.000	1.000	0.000	0.000	2.333	0.069	0.015
PredObs6	KORONEIKI	0.000	1.000	0.000	0.000	2.454	0.129	-0.142
PredObs7	KORONEIKI	0.000	1.000	0.000	0.000	2.284	0.071	-0.220
PredObs8	KORONEIKI	0.003	0.997	0.000	0.000	-0.091	-0.016	0.303
PredObs9	KORONEIKI	0.000	1.000	0.000	0.000	1.456	-0.389	-0.884
PredObs10	KORONEIKI	0.000	1.000	0.000	0.000	1.654	-0.181	-0.701
PredObs11	KORONEIKI	0.000	1.000	0.000	0.000	1.636	-0.315	-1.112
PredObs12	KORONEIKI	0.001	0.999	0.000	0.000	0.841	-0.270	-1.298
PredObs13	KORONEIKI	0.000	1.000	0.000	0.000	2.745	0.471	0.233
PredObs14	KORONEIKI	0.000	1.000	0.000	0.000	1.587	-1.637	-0.840
PredObs15	KORONEIKI	0.000	1.000	0.000	0.000	2.183	-0.042	-0.105
PredObs16	KORONEIKI	0.000	1.000	0.000	0.000	3.048	-0.114	-0.211
PredObs17	KORONEIKI	0.000	1.000	0.000	0.000	2.176	-1.052	-1.201
PredObs18	KORONEIKI	0.000	1.000	0.000	0.000	3.728	-0.952	0.508
PredObs19	KORONEIKI	0.000	1.000	0.000	0.000	4.951	-1.179	1.310
PredObs20	KORONEIKI	0.000	1.000	0.000	0.000	1.652	-0.035	-0.705
PredObs21	KORONEIKI	0.000	1.000	0.000	0.000	3.607	-0.306	0.862
PredObs22	KORONEIKI	0.000	1.000	0.000	0.000	5.210	0.379	2.595
PredObs23	KORONEIKI	0.000	1.000	0.000	0.000	4.337	-0.136	0.173
PredObs24	KORONEIKI	0.000	1.000	0.000	0.000	4.522	0.076	0.159
PredObs25	KORONEIKI	0.000	1.000	0.000	0.000	3.866	-0.081	0.375
PredObs26	KORONEIKI	0.000	1.000	0.000	0.000	3.756	-1.490	0.980
PredObs27	KORONEIKI	0.000	1.000	0.000	0.000	3.751	-0.960	-0.270
PredObs28	MEGARITIKI	0.000	0.000	0.000	1.000	-5.990	-3.899	-0.487
PredObs29	MEGARITIKI	0.000	0.000	0.000	1.000	-6.370	-4.257	0.093
PredObs30	MEGARITIKI	0.000	0.000	0.000	1.000	-6.858	-3.069	-1.055
PredObs31	MEGARITIKI	0.000	0.000	0.000	1.000	-4.197	-2.228	0.103
PredObs32	MEGARITIKI	0.000	0.000	0.000	1.000	-6.110	-4.427	-1.165
PredObs33	MEGARITIKI	0.000	0.000	0.000	1.000	-5.092	-3.883	-0.165
PredObs34	MEGARITIKI	0.000	0.000	0.000	1.000	-5.827	-3.066	1.477
PredObs35	AMFISSIS	1.000	0.000	0.000	0.000	-3.181	2.979	-2.958
PredObs36	AMFISSIS	1.000	0.000	0.000	0.000	-1.237	3.080	-1.690
PredObs37	AMFISSIS	1.000	0.000	0.000	0.000	-1.758	3.259	-1.025
PredObs38	AMFISSIS	0.726	0.274	0.000	0.000	0.665	2.565	-0.789
PredObs39	AMFISSIS	1.000	0.000	0.000	0.000	-0.028	3.896	-1.263
PredObs40	MEGARITIKI	0.000	0.000	0.000	1.000	-2.099	-3.138	0.689
PredObs41	AMFISSIS	1.000	0.000	0.000	0.000	-0.259	3.899	-1.635

Table S12. Results for the prediction samples of quadratic discriminant analysis.

Observations	Predicted class	Pr(AMFIS-SIS)	Pr(KORO-NEIKI)	Pr(MANAKI)	Pr(MEGARI-TIKI)	F1	F2	F3
PredObs1	MEGARITIKI	0.012	0.000	0.000	0.988	-3.910	4.088	1.579
PredObs2	MANAKI	0.000	0.000	1.000	0.000	-4.695	4.220	3.079
PredObs3	MEGARITIKI	0.000	0.092	0.000	0.908	-2.851	3.326	2.466
PredObs4	MANAKI	0.000	0.000	1.000	0.000	-3.889	4.613	2.921
PredObs5	KORONEIKI	0.000	1.000	0.000	0.000	2.333	0.069	0.015
PredObs6	KORONEIKI	0.000	1.000	0.000	0.000	2.454	0.129	-0.142
PredObs7	KORONEIKI	0.000	1.000	0.000	0.000	2.284	0.071	-0.220
PredObs8	KORONEIKI	0.000	0.785	0.000	0.215	-0.091	-0.016	0.303
PredObs9	KORONEIKI	0.000	1.000	0.000	0.000	1.456	-0.389	-0.884
PredObs10	KORONEIKI	0.000	1.000	0.000	0.000	1.654	-0.181	-0.701
PredObs11	KORONEIKI	0.000	1.000	0.000	0.000	1.636	-0.315	-1.112
PredObs12	KORONEIKI	0.000	1.000	0.000	0.000	0.841	-0.270	-1.298
PredObs13	KORONEIKI	0.000	1.000	0.000	0.000	2.745	0.471	0.233
PredObs14	KORONEIKI	0.000	1.000	0.000	0.000	1.587	-1.637	-0.840
PredObs15	KORONEIKI	0.000	1.000	0.000	0.000	2.183	-0.042	-0.105
PredObs16	KORONEIKI	0.000	1.000	0.000	0.000	3.048	-0.114	-0.211
PredObs17	KORONEIKI	0.000	1.000	0.000	0.000	2.176	-1.052	-1.201
PredObs18	KORONEIKI	0.000	1.000	0.000	0.000	3.728	-0.952	0.508
PredObs19	KORONEIKI	0.000	1.000	0.000	0.000	4.951	-1.179	1.310
PredObs20	KORONEIKI	0.000	1.000	0.000	0.000	1.652	-0.035	-0.705
PredObs21	KORONEIKI	0.000	1.000	0.000	0.000	3.607	-0.306	0.862
PredObs22	KORONEIKI	0.000	1.000	0.000	0.000	5.210	0.379	2.595
PredObs23	KORONEIKI	0.000	1.000	0.000	0.000	4.337	-0.136	0.173
PredObs24	KORONEIKI	0.000	1.000	0.000	0.000	4.522	0.076	0.159
PredObs25	KORONEIKI	0.000	1.000	0.000	0.000	3.866	-0.081	0.375
PredObs26	KORONEIKI	0.000	1.000	0.000	0.000	3.756	-1.490	0.980
PredObs27	KORONEIKI	0.000	1.000	0.000	0.000	3.751	-0.960	-0.270
PredObs28	MEGARITIKI	0.000	0.000	0.000	1.000	-5.990	-3.899	-0.487
PredObs29	MEGARITIKI	0.000	0.000	0.000	1.000	-6.370	-4.257	0.093
PredObs30	MEGARITIKI	0.000	0.000	0.000	1.000	-6.858	-3.069	-1.055
PredObs31	MEGARITIKI	0.000	0.000	0.000	1.000	-4.197	-2.228	0.103
PredObs32	MEGARITIKI	0.000	0.000	0.000	1.000	-6.110	-4.427	-1.165
PredObs33	MEGARITIKI	0.000	0.000	0.000	1.000	-5.092	-3.883	-0.165
PredObs34	MEGARITIKI	0.000	0.000	0.000	1.000	-5.827	-3.066	1.477
PredObs35	AMFISSIS	0.997	0.000	0.000	0.003	-3.181	2.979	-2.958
PredObs36	AMFISSIS	1.000	0.000	0.000	0.000	-1.237	3.080	-1.690
PredObs37	AMFISSIS	1.000	0.000	0.000	0.000	-1.758	3.259	-1.025
PredObs38	AMFISSIS	0.999	0.001	0.000	0.000	0.665	2.565	-0.789
PredObs39	AMFISSIS	1.000	0.000	0.000	0.000	-0.028	3.896	-1.263
PredObs40	MEGARITIKI	0.000	0.000	0.000	1.000	-2.099	-3.138	0.689
PredObs41	AMFISSIS	1.000	0.000	0.000	0.000	-0.259	3.899	-1.635

Table S13. Confusion matrix for the cross-validation results of linear and quadratic discriminant analysis.

From \ To	AMFISSIS	KORONEIKI	MANAKI	MEGARITIKI	Total	% Correct
Linear discriminant analysis						
AMFISSIS	24	0	0	0	24	100.00%
KORONEIKI	0	92	0	0	92	100.00%
MANAKI	0	0	14	0	14	100.00%

MEGARITIKI	0	0	0	28	28	100.00%
Total	24	92	14	28	158	100.00%
			Quadratic discriminant analysis			
AMFISSIS	23	1	0	0	24	95.83%
KORONEIKI	0	91	0	1	92	98.91%
MANAKI	0	0	14	0	14	100.00%
MEGARITIKI	0	1	0	27	28	96.43%
Total	23	93	14	28	158	98.10%

Table S14. Confusion matrix for the prediction results of linear and quadratic discriminant analysis.

From \ To	AMFISSIS	KORONEIKI	MANAKI	MEGARITIKI	Total	% Correct
			Linear discriminant analysis			
AMFISSIS	6	0	0	1	7	97.56%
KORONEIKI	0	23	0	0	23	100.00%
MANAKI	0	0	4	0	4	100.00%
MEGARITIKI	0	0	0	7	7	100.00%
Total	6	23	4	8	41	97.56%
			Quadratic discriminant analysis			
AMFISSIS	6	0	0	1	7	92.68%
KORONEIKI	0	23	0	0	23	100.00%
MANAKI	0	0	2	2	4	100.00%
MEGARITIKI	0	0	0	7	7	100.00%
Total	6	23	4	8	41	92.68%
